# Amphiphilic Aminated Derivatives of [60]Fullerene as Potent Inhibitors of Tumor Growth and Metastasis

**DOI:** 10.1002/advs.202201541

**Published:** 2022-08-28

**Authors:** Jiawei Huo, Jie Li, Yang Liu, Libin Yang, Xinran Cao, Chong Zhao, Yicheng Lu, Wei Zhou, Shumu Li, Jianan Liu, Jiao Li, Xing Li, Jing Wan, Rui Wen, Mingming Zhen, Chunru Wang, Chunli Bai

**Affiliations:** ^1^ Beijing National Laboratory for Molecular Sciences Key Laboratory of Molecular Nanostructure and Nanotechnology Institute of Chemistry Chinese Academy of Sciences Beijing 100190 China; ^2^ University of Chinese Academy of Sciences Beijing 100049 China; ^3^ School of Pharmacy Guizhou Medical University Guian New District Guizhou 550025 China

**Keywords:** amphipathic aminated [60]fullerene, cell cycle arrest, cell mobility suppression, mesenchymal–epithelial transition, protein target

## Abstract

Malignant proliferation and metastasis are the hallmarks of cancer cells. Aminated [70]fullerene exhibits notable antineoplastic effects, promoting it a candidate for multi‐targeted cancer drugs. It is an urgent need to reveal the structure–activity relationship for antineoplastic aminated fullerenes. Herein, three amphiphilic derivatives of [60]fullerene with clarified molecular structures are synthesized: TAPC‐4, TAPC‐3, and TCPC‐4. TAPC‐4 inhibits the proliferation of diverse tumor cells via G0/G1 cell cycle arrest, reverses the epithelial–mesenchymal transition, and abrogates the high mobility of tumor cells. TAPC‐4 can be excreted from the organism and achieves an in vivo inhibition index of 75.5% in tumor proliferation and 87.5% in metastatic melanoma with a wide safety margin. Molecular dynamics simulations reveal that the amphiphilic molecular structure and the ending amino groups promote the targeting of TAPC‐4 to heat shock protein Hsp90‐beta, vimentin, and myosin heavy chain 9 (MYH9), probably resulting in the alteration of cyclin D1 translation, vimentin expression, and MYH9 location, respectively. This work initially emphasizes the dominant role of the amphiphilic structure and the terminal amino moieties in the antineoplastic effects of aminated fullerenes, providing fundamental support for their anti‐tumor drug development.

## Graphical abstract

TAPC‐4, an amphiphilic aminated [60]fullerene with a well‐defined structure, prevents tumor growth and metastasis by blocking the cell cycle in G0/G1 phase, reversing the epithelial‐mesenchymal transition process, and regulating the localization of myosin heavy chain 9, which may be achieved by binding against specific proteins.

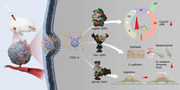

## 1 Introduction

Cancer is the second leading cause of death and arises tremendous physical, emotional, and financial burdens globally. Sustaining proliferation, malignant invasion, and metastasis are the primary hallmarks of cancer cells.^[^ ^1^ ^]^ Fullerene derivatives exhibit notable antineoplastic effects and a wide margin of safety.^[^ ^2^ ^]^ Hydroxylated fullerenes inhibit tumor growth and metastasis via remodeling tumor microenvironment (TME), including antioxidant defense, immune activation, angiogenesis disruption, elimination of cancer stem cells, and regulation of  the expression and activity of oncoproteins (e.g., MMP‐9, HDAC1, MTA1, collagen, actin).^[^ ^3^ ^]^ Hydroxylated fullerenes consist of various isomers with different molecular structures, probably resulting in diverse therapeutic mechanisms.^[^ ^4^ ^]^ Compared with hydroxylated fullerenes targeting TME, functional fullerenes with novel structures can directly inhibit cancer cell proliferation via cell cycle arrest or autophagy regulation.^[^ ^5^ ^]^ In previous work, we designed multiple ethylenediamine (EDA) modified [70]fullerene (abbreviated as C_70_‐EDA) with EDA moieties in the number range of 6–11 and explored its protein targets.^[^ ^6^ ^]^ C_70_‐EDA binds against tumor‐specific myosin heavy chain 9 (MYH9), transporting MYH9 from the cytoplasm to the cell edge, abrogating metastasis‐associated cell migration, and reversing epithelial‐mesenchymal transition (EMT). In addition, C_70_‐EDA can target mRNA‐binding proteins and mRNA transport‐associated proteins for post‐transcriptional regulations, activating autophagic flux and inducing G0/G1 cell cycle arrest to inhibit cancer cells directly.^[^ ^7^ ^]^ The inhibition of tumor proliferation and metastasis via targeting tumor‐specific regulators promotes C_70_‐EDA a promising candidate for anti‐tumor drugs. It is essential to establish the relationship between molecule structure and antineoplastic activity for further pharmaceutical development. However, the ambiguous molecular structure of C_70_‐EDA makes it challenging to explore the critical pharmacophore required for its antineoplastic effect.

Herein, three amphiphilic derivatives of [60]fullerene with explicit molecular structures were synthesized in high purities: tetra[4‐(amino)piperidin‐1‐yl]C_60_ epoxide (TAPC‐4), tri[4‐(amino)piperidin‐1‐yl]‐hydro‐C_60_ epoxide (TAPC‐3), and tetra[4‐(carboxyl)piperidin‐1‐yl]C_60_ epoxide (TCPC‐4). TAPC‐4 can directly inhibit tumor cell proliferation via G0/G1 cell cycle arrest and abrogate tumor metastasis by suppressing cell mobility and reversing the EMT. Compared with TAPC‐4, TAPC‐3 exhibits a similar high inhibitory effect, but TCPC‐4 gets a lower antineoplastic efficacy. Drug target screening unveiled that TAPC‐4 could bind against heat shock protein Hsp90‐beta (Hsp90*β*), vimentin, and MYH9 to regulate the cell cycle, EMT, and cell mobility, respectively. Molecular docking and molecular dynamics (MD) simulations were performed to explore the binding mode of TAPC‐4 to Hsp90*β*, vimentin, and MYH9 and further elucidate the special antineoplastic mechanism of TAPC‐4.

## 2 Results and Discussion

### 2.1 Amphiphilic Derivatives of [60]Fullerene Inhibit Tumor Proliferation In Vitro

**Figure 1:**
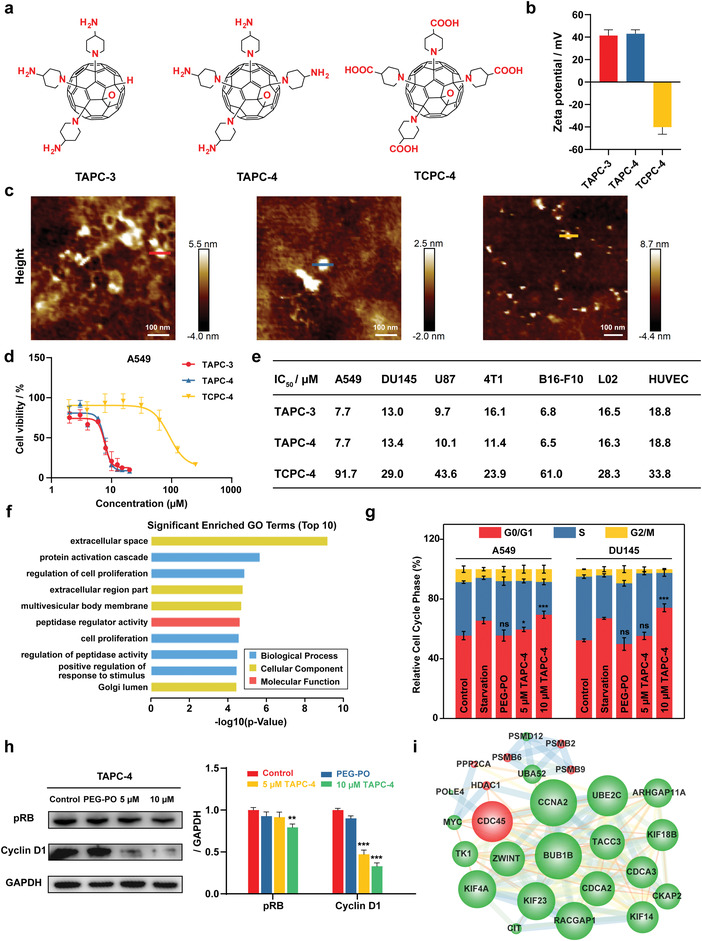
TAPC abrogates the proliferation of various cancer cells via cell cycle arrest. a) Molecular structure, b) zeta potential (*n* = 3), and c) AFM images of TAPC‐3, TAPC‐4, and TCPC‐4. d) The viability of A549 cells separately treated with TAPC‐3, TAPC‐4, and TCPC‐4 at gradient concentrations for 24 h (*n* = 6). e) List of IC_50_ values in diverse cell lines. f) The top 10 GO terms with the lowest *p*‐value. Enrichment analysis of significantly different proteins. g) Quantification of A549 and DU145 cells in G0/G1, S, or G2/M phase after TAPC‐4 treatment (*n* = 3). G0/G1 arrest was induced under serum‐starved conditions as a positive control. h) Left: Expression of cyclin D1 and pRB in A549 cells treated with TAPC‐4 for 24 h. GAPDH as a loading control. Right: Quantifications (*n* = 3). i) PPI network of the proteins associated with the cell cycle. The nodes and edges represent protein and protein‐protein interaction. The node size indicates its degree, and up‐regulation and down‐regulation are separately labeled red and green (Mean ± SEM; Student's *t*‐test, *p < 0.05, **p < 0.01, and ***p < 0.001).

The amphiphilic derivatives of [60]fullerene, TAPC‐4, TAPC‐3, and TCPC‐4, were synthesized following the reported method (**Figure** **1**a; Figure S1, Supporting Information).^[^ ^8^ ^]^ In brief, TAPC‐4 and TAPC‐3 protected by tert‐butoxycarbonyl (Boc) and tert‐butyl ester of TCPC were prepared by one‐step synthesis on a multigram scale. As the synthesis reaction proceeded, C_60_ was continuously consumed and undetectable after 48 h, and the limit of detection for C_60_ was 0.3 µg mL^−1^ (Figure S2, Supporting Information). The synthesized intermediates were characterized by high‐performance liquid chromatography (HPLC), nuclear magnetic resonance (NMR) spectroscopy, and electrospray ionization mass spectrometry (ESI‐MS) (Figures S3–S5, Supporting Information). There are two possible isomers of Boc‐protected TAPC‐3, and the thermodynamically favored isomer was screened via theoretical simulation (Table S1 and Figure S6, Supporting Information). Subsequently, Boc and tert‐butyl groups were removed by trifluoroacetic acid (TFA) in CHCl_3_ to obtain TAPC‐3, TAPC‐4, and TCPC‐4, respectively. Finally, hydrochloride of TAPC‐3 and TAPC‐4 and sodium salt of TCPC‐4 with excellent aqueous solubility were prepared via ion exchange and characterized by liquid chromatography‐electrospray ionization mass spectrometry (LC–ESI/MS) and NMR (Figures S7–S10, Supporting Information). Water‐soluble TAPC‐3, TAPC‐4, and TCPC‐4 with purities above 95% were utilized for further investigation. The average zeta potentials of TAPC‐3, TAPC‐4, and TCPC‐4 are 41.4, 43.1, and −40.1 mV in an aqueous solution, respectively (Figure 1b). The ammonium moieties of TAPC‐3 and TAPC‐4 result in their positive zeta potentials, and the carboxylate groups of TCPC‐4 account for its negative zeta potential. According to the height signal map obtained by atom force microscopy (AFM), the statistical particle size distributions for TAPC‐3, TAPC‐4, and TCPC‐4 are 8.2 ± 4.3, 6.3 ± 3.5, and 12.7 ± 5.0 nm, respectively (Figure 1c; Figure S11, Supporting Information). Amphiphilic fullerene derivatives self‐assemble to form nanoclusters in aqueous solutions.^[^ ^9^ ^]^ Correspondingly, TAPC‐3, TAPC‐4, and TCPC‐4 assemble into nanoclusters of 126.1, 142.6, and 161.5 nm in hydrodynamic size, respectively (Figure S12a, Supporting Information). Protonation of amino groups under acidic conditions (pH ≤ 5) or heating (temperature ≥ 60 °C) promotes the breakdown of TAPC self‐assembled nanoclusters into clusters smaller than 10 nm (Figure S12b,c, Supporting Information). Positively charged aminated fullerenes tend to aggregate in physiological conditions. As reported previously, phosphorylated polyethylene glycol (PEG‐PO) is an optimum stabilizing ligand to attenuate the aggregation of aminated fullerenes and exhibit a negligible effect on their antineoplastic efficacy.^[^ ^10^ ^]^ Herein, TAPC‐3 and TAPC‐4 were protected from self‐aggregation in biological assays via coating with a tenfold molar ratio of PEG‐PO (*M*_w_ = 1900) (Figure S13, Supporting Information).

The antineoplastic effects of amphiphilic C_60_ derivatives were evaluated in various cancer cell lines, including A549 lung cancer cell, DU145 prostate cancer cell, U87 primary glioblastoma cell, 4T1 breast cancer cell, and B16‐F10 melanoma cells. The half inhibitory concentration (IC_50_) against cancer cells was calculated. The IC_50_ values of PEG‐PO‐coated TAPC‐3 and TAPC‐4 in various cancer cells are approximately 10 µm, and that of TCPC ranges from 20 to 90 µm (Figure 1d,e; Figure S14, Supporting Information). In contrast, the pristine C_60_ coated with cyclodextrin exhibits no cytotoxicity at concentrations up to 100 µm (Figure S15, Supporting Information). PEG‐PO at maximum concentrations exhibits negligible cytotoxicity. TAPC‐3 and TAPC‐4, aminated derivatives of C_60_ with different amino moieties, exhibit comparable inhibition in tumor cell proliferation. Still, TCPC‐4, carboxylated C_60_ with the same amphiphilic structure, gets a significantly reduced antineoplastic activity. The IC_50_ values of TAPC‐3 and TAPC‐4 in L‐02 human liver cells and HUVEC human umbilic vein endothelial cells increase to approximately 16 µm, revealing their reduced inhibitory effects on normal cells. As TAPC‐4 gets notable anti‐tumor efficacy and a much higher yield than TAPC‐3, TAPC‐4 was chosen for further in vitro and in vivo assays.

We carried out a comprehensive proteomics analysis to explore the antineoplastic mechanism of TAPC‐4. A549 cells were separately treated with TAPC‐4 (10 µm) and PBS (NC) for 24 h with three repeats. A total of 6487 proteins have been identified and quantified. The difference in protein expression induced by TAPC‐4 is revealed via correlation analysis and hierarchical clustering (Figure S16, Supporting Information). Proteins with a fold change >1.5 and *p*‐value < 0.05 are considered differentially expressed with significance. There are 508 proteins with significantly different expressions, including 267 up‐regulated and 241 down‐regulated (Figure S17, Supporting Information). KEGG pathway enrichment and GO annotation were performed on significantly different proteins (Figures S18–S21, Supporting Information). The top ten GO terms with the minimum *p*‐values have been obtained (Figure 1f). Biological processes termed “regulation of cell proliferation” and “cell proliferation” are notable in the GO annotation, indicating TAPC‐4 could probably alter the proliferation of cancer cells. Consequently, the cell cycle was quantitively monitored by flow cytometry. A549 and DU145 cells were incubated with PEG‐PO coated TAPC‐4 at concentrations up to 10 µm for 24 h, and the proportion of cells in the G0/G1 phase was raised from 50% to 70% (Figure 1g; Figure S22, Supporting Information). As a positive control, cells were cultured in a serum‐free medium to induce G0/G1 arrest. PEG‐PO did not affect the cell cycle, revealing that it was TAPC‐4 causing the G0/G1 arrest. The complex of cyclin D (CCND) and CDK4/6 is critical in regulating the G1/S transition checkpoint. The activation of the CCND1‐CDK4/6 complex can phosphorylate retinoblastoma (RB), releasing RB‐bound transcription factors (e.g., E2F1, E2F2, and E2F3) to drive the cell cycle into the S phase.^[^ ^11^ ^]^ TAPC‐4 induced the downregulation of cyclin D1 and phosphorylated RB (pRB) in a dose‐dependence (Figure 1h). Protein–protein interaction (PPI) networks on the differential proteins associated with cell cycle regulation were generated (Figure 1i). Interestingly, most of these proteins were strongly responsible for the production of cell cycle proteins.^[^ ^12^ ^]^ For instance, silencing UBE2C suppressed cell proliferation by inducing G1/S arrest mediated by downregulation of cyclin D1 in CFPAC‐1 and PANC‐1 cells, and cyclin D1, Bcl‐2, and survivin could be reduced by down‐regulation of CKAP2 in osteosarcoma cells. The decrease in their expression may lead to the reduction of cyclin D1 protein content.

Therefore, water‐soluble aminated and carboxylated derivatives of C_60_ with clarified molecular structures are readily synthesized with high purities. TAPC‐3 and TAPC‐4 exhibit comparably higher antineoplastic efficacies than TCPC‐4. TAPC‐3 gets two isomers and a much lower yield, suggesting the prior application of TAPC‐4 in cancer therapy. As indicated by proteomic analysis, TAPC‐4 blocks the cell cycle in the G0/G1 phase by down‐regulating cyclin D1 and subsequently reducing the phosphorylation of RB. C_70_‐EDA also induced G0/G1 arrest and got IC_50_ values of ≈20 µm in our previous work. TAPC with clarified molecular structure and superior anti‐tumor activity is more suitable for drug development.

### 2.2 TAPC‐4 Abrogates Cancer Cell Migration and Induces MET

Increased mobility and EMT are the hallmarks of cancer metastasis. EMT is a reversible biological process: epithelial cells lose intercellular junctions, apicobasal polarity, and immobility and redirect to mesenchymal phenotype with the enhanced ability for migration and invasion.^[^ ^13^ ^]^ Vimentin, Snail, and N‐cadherin serve as critical indicators of EMT.^[^ ^14^ ^]^ Vimentin is a type III intermediate filament generally expressed in mesenchymal cells but up‐regulated during cancer metastasis.^[^ ^15^ ^]^ Vimentin plays a pivotal role in supporting the migratory machinery's mechanical integrity, directional force generation, and extracellular attachment.^[^ ^16^ ^]^ Snail is a zinc finger transcriptional repressor facilitating EMT by suppressing specific genes.^[^ ^17^ ^]^ E‐cadherin, a canonical epithelial marker, is a transmembrane glycoprotein maintaining cell‐to‐cell adhesion via association with the actin cytoskeleton.^[^ ^18^ ^]^ The downregulation of E‐cadherin and the concomitant upregulation of N‐cadherin enhances migratory and invasive traits.

**Figure 2:**
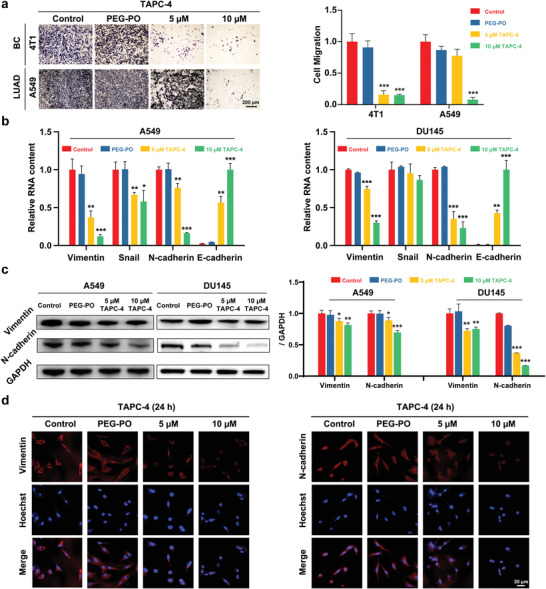
TAPC‐4 inhibits cancer cell migration and reverses the EMT. a) Left: Representative images of migrated cancer cells under TAPC‐4 treatments. Right: Quantifications (*n* = 4). LUAD: lung adenocarcinoma, BC: breast cancer. b) Relative mRNA levels of EMT markers (N‐cadherin, E‐cadherin, Vimentin, and Snail) in A549 and DU145 cells treated by TAPC‐4 for 24 h (*n* = 4). c) Left: Protein levels of mesenchymal markers (Vimentin and N‐cadherin) by immunoblotting. Right: Quantifications (*n* = 3). d) Representative immunofluorescence images of N‐cadherin and Vimentin in A549 cell lines treated with TAPC‐4 for 24 h (*n* = 3). The scale bar is 30 µm (Mean ± SEM; Student's *t*‐test, *p < 0.05, **p < 0.01, and ***p < 0.001).

Quantitative evaluation of cancer cell mobility was carried out by in vitro transwell assay: A549 and 4T1 cells pre‐treated in a serum‐free medium were added to the upper chamber of microporous membrane, and cells transferred to the down chamber containing medium with 20% FBS were stained with crystal violet and counted. TAPC‐3 and TAPC‐4 significantly diminished the mobility of tumor cells. The number of cells penetrating the porous membrane dropped by up to 90% after TAPC‐4 treatment for 6 h (**Figure** **2**a; Figure S23, Supporting Information). The effective concentration of TAPC‐4 to inhibit tumor cell migration is approximately 5 µm, much lower than that of hydroxylated fullerenes (≈50 µm).^[^ ^2^, ^3^ ^]^ Meanwhile, the alteration of EMT by TAPC‐4 was investigated. TAPC‐4 increased the expression of epithelial markers (E‐cadherin) and reduced the expression of mesenchymal markers (vimentin, Snail, N‐cadherin) at mRNA levels in A549 and DU145 cells (Figure 2b). Correspondingly, the protein expression of vimentin and N‐cadherin in A549 and DU145 cells was reduced by TAPC‐4 in a dose‐dependence, as revealed by Western blotting (Figure 2c) and immunofluorescence (Figure 2d; Figure S24, Supporting Information). Consequently, TAPC‐4 promoted mesenchymal‐epithelial transition (MET), the reversed process of EMT.

As a proto‐oncogene protein, Myc got down‐regulated and ranked at the top in the PPI network of total proteins with significantly different expressions (Figure S25, Supporting Information). Myc regulates the expression of vimentin and associated pathways for EMT induction and cell migration.^[^ ^19^ ^]^ Downregulation of vimentin reduces mRNA and protein expression of Snail via a feedback loop.^[^ ^20^ ^]^ Snail is a transcription factor that regulates EMT by repressing E‐cadherin expression to reduce cellular junctions and increase cell migration.^[^ ^21^ ^]^ Taken together, TAPC‐4 reduced the expression of vimentin at both mRNA and protein levels, which may be partly associated with the downregulation of Myc. The low level of vimentin probably results in the downregulation of Snail, thus repealing the inhibition of E‐cadherin expression.

### 2.3 TAPC‐4 Targets Specific Tumor Regulators

**Figure 3:**
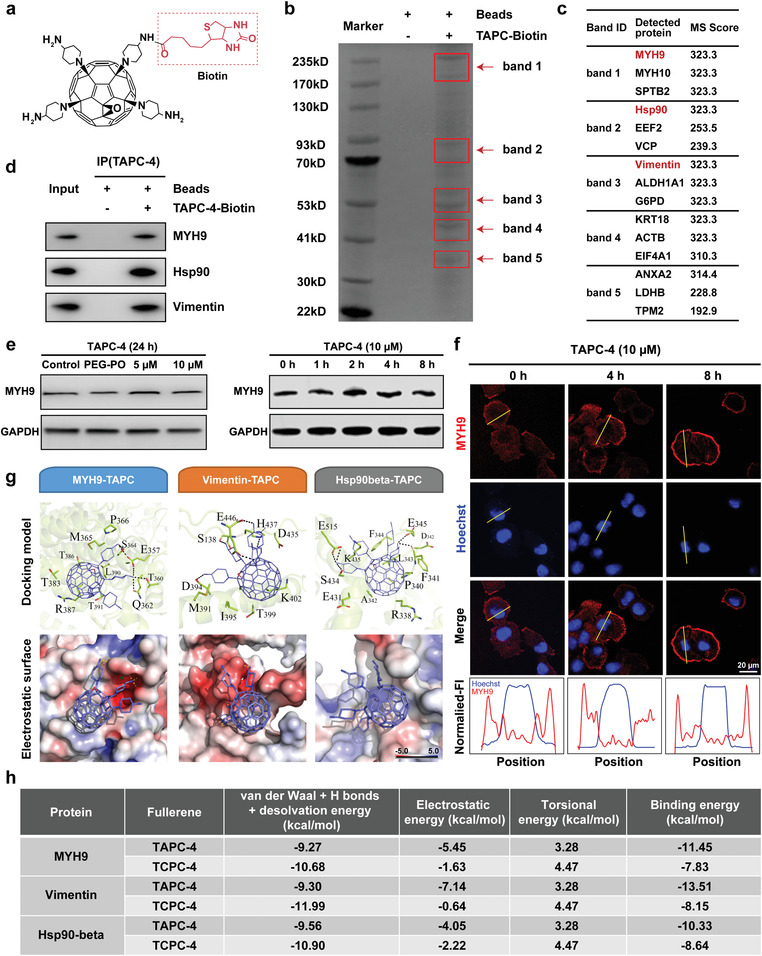
Enrichment and identification of protein targets. a) Molecular structure of thermodynamically favored biotinylated TAPC‐4 isomer. b) Coomassie brilliant blue‐stained SDS‐PAGE utilized to separate the proteins enriched by biotinylated TAPC‐4 from A549 cell lysate. Specific bands for further MS identification were assigned red arrows and termed band 1 to band 5 by molecular weight. c) List of the top three target proteins with the highest MS score in each band. d) Immunoprecipitation (IP) of MYH9, vimentin, and Hsp90*β* by biotinylated TAPC‐4 from A549 cell lysate. e) MYH9 expression in A549 cells treated with TAPC‐4 in concentration and time gradients (*n* = 3). f) Representative immunofluorescence image of MYH9 protein in DU145 cells treated with TAPC‐4 in time gradient. MYH9 and cell nucleus were labeled with Alexa 647 (Red) and Hoechst (blue) separately. The normalized fluorescence of MYH9 and Hoechst along the yellow line was shown below the corresponding images (*n* = 3). The scale bar is 30 µm. g) Top: The optimal docking model of TAPC‐4 to MYH9, vimentin, and Hsp90*β*. TAPC‐4 and amino acids are shown in blue and green, respectively. Bottom: The binding mode of TAPC‐4 in MYH9, vimentin, and Hsp90*β*. The active site pocket is displayed as an electrostatic surface. h) Components of the calculated binding free energies.

To clarify the antineoplastic mechanism of TAPC‐4, we screened the potential protein targets of TAPC‐4 by pull‐down assay. In brief, TAPC‐4 was labeled with D‐biotin on the primary amino group, and two isomers of biotinylated TAPC‐4 were obtained with comparable yields and characterized by LC‐MS (Figure S26, Supporting Information). The two isomers get very close heat of formations, as revealed by theoretical simulations (Table S2 and Figure S27, Supporting Information). The one with comparatively high thermodynamic stability is shown (**Figure** **3**a). It is not easy to separate the two isomers with similar structures, so a mixture was utilized for the pull‐down assay. About 10 nmol of biotinylated TAPC‐4 was adequately mixed with A549 cell lysate, and the complexes of biotinylated TAPC‐4 and its binding proteins were captured with streptavidin magnetic beads. The “pulled down” proteins were eluted for SDS‐PAGE separation and stained by Coomassie brilliant blue (Figure 3b). To eliminate the unspecific binding proteins, the control group was treated without biotinylated TAPC‐4 but only streptavidin magnetic beads. Five notable additional protein bands compared to the control were digested for protein identification by LC‐MS (Figure 3c). The top ten assigned possible proteins ranked by MS score in each band were screened for further analysis (Table S3, Supporting Information).

Hsp90*β*, vimentin, and MYH9, with the highest MS score in bands 1–3 and critical roles in tumor cell proliferation, mobility, and EMT process, are the possible targets of TAPC‐4 for tumor inhibition (Figure 3c). The specific binding interactions between TAPC‐4 and Hsp90*β*, vimentin, and MYH9 were verified by immunoprecipitation assay (Figure 3d). Hsp90 is involved in protein folding and maturation and plays an essential role in tumor survival, as many Hsp90 clients are oncogenic kinases such as CDK4, EGFR, HER2, AKT, MET, MEK, and BCR‐ABL.^[^ ^22^ ^]^ Hsp90 inhibitors can induce simultaneous proteasomal degradation of a wide range of client oncoproteins and thus abolish the malignant proliferation and metastasis of cancer cells, so various Hsp90 inhibitors are currently evaluated in ≈50 clinical trials.^[^ ^23^ ^]^ Hsp90 inhibitors induce the ubiquitin‐dependent degradation of AKT, inhibiting the translation of cyclin D1 via the PI3K/AKT pathway.^[^ ^24^ ^]^ TAPC‐4 induces the downregulation of cyclin D1 expression and subsequent G0/G1 cell cycle arrest (Figure 1h), indicating that the specific interaction of TAPC‐4 with Hsp90 probably inhibits the translation of cyclin D1 via PI3K/AKT pathway. Vimentin appears to be at the center of the EMT/MET pathway, controlling the cancer cells’ plasticity. Vimentin‐specific inhibitors reduce the protein level of vimentin via caspase‐3 mediated proteolysis.^[^ ^25^ ^]^ Consequently, TAPC‐4 binds against vimentin and downregulates vimentin expression (Figure 2b–d), probably through the proteolysis pathway. Non‐muscle myosin IIA (NM‐IIA), consisting of a homodimer of MYH9, two regulatory light chains, and two essential light chains, tends to self‐assemble into filaments supporting cell migration.^[^ ^26^ ^]^ C_70_‐(EDA)*_n_* binds against MYH9 to regulate the subcellular distribution of MYH9 and inhibits tumor cell migration, probably attributing to the disassembly of NM‐IIA filaments.^[^ ^6^ ^]^ Correspondingly, the binding of TAPC‐4 to MYH9 did not change its protein expression (Figure 3e) but triggered the transport of MYH9 from the cytoplasm to the cell edge, thus abrogating the mobility of tumor cells (Figure 3f).

To unveil the molecular mechanisms underlying the superior antineoplastic efficacy of TAPC‐4 compared with TCPC‐4, the binding mode of TAPC‐4 and TCPC‐4 to Hsp90*β*, vimentin, and MYH9 was explored by molecular docking and MD simulations, respectively. The optimized molecular structures of TAPC‐4 and TCPC‐4 were obtained by theoretical simulation (Figure S28, Supporting Information). The crystal structure of the human Hsp90*β* protein (PDB code 5FWK) from Protein Data Bank and 3D protein structures for amino acid sequences of vimentin and MYH9 derived from AlphaFold Bank were used as the receptors in the docking model. The top 100 binding sites with minimal energy were screened via molecular docking (Figure S29, Supporting Information). Then, the docked complex with the lowest binding energy was selected for further MD simulations and carried out in the explicit water environment for 80 ns. The root‐mean‐square deviation (RMSD) values for the TAPC‐4 complexes gradually became stable after 40 ns (Figure S30, Supporting Information), and that for the TCPC‐4 complexes tended to converge after 20 ns (Figure S31, Supporting Information). Average structures of the equilibrated ligand‐receptor complexes were generated for free binding energy calculation. TAPC‐4 and TCPC‐4 bind to the hydrophobic pocket of Hsp90*β*, primarily attributing to the hydrophobic interactions between the fullerene carbon cage and the pocket (Figure 3g; Figure S32, Supporting Information). TAPC‐4 is closely embraced by hydrophobic residues P340, F341, A342, L343, and F344 via van der Waals interactions, and TCPC‐4 is held by residues R338, A339, and F341. The amino groups of TAPC‐4 formed multiple hydrogen bonds and electrostatic interactions with hydrophilic residues F341, E345, S434, and E515 in the site pocket of Hsp90*β*, promoting TAPC‐4 to get more negative electrostatic energy than TCPC‐4 and thus the stronger binding to Hsp90*β* (Figure 3h). Due to the relatively large molecular size, TAPC‐4 and TCPC‐4 do not get into the internal pocket of vimentin and MYH9 but bind to their surface minor groove regions. Compared with TCPC‐4, TAPC‐4 has close free energies of van der Waals interactions, hydrogen bonds, and desolvation and torsional energies but more negative electrostatic energies, resulting in the stronger binding of TAPC‐4 to vimentin and MYH9. Notably, the free binding energy of TAPC‐4 to vimentin is the most negative, indicating vimentin is probably the prior target of TAPC‐4. Overall, amphiphilic TAPC‐4 and TCPC‐4 both can bind to Hsp90*β*, vimentin, and MYH9 primarily through hydrophobic interactions of fullerene carbon cages with the protein “pockets,” and the terminal amino moieties of TAPC‐4 can form solid electrostatic interactions with the hydrophilic amino acid residues of the pockets to enhance its binding to the target proteins compared with TCPC‐4. Consequently, molecular docking and MD simulations indicate that the amphiphilic molecular structure and the terminal amino groups enable the binding of TAPC‐4 against Hsp90*β*, vimentin, and MYH9, probably resulting in its high antineoplastic efficacy in vitro.

### 2.4 Distribution and Excretion of TAPC‐4 In Vivo

**Figure 4:**
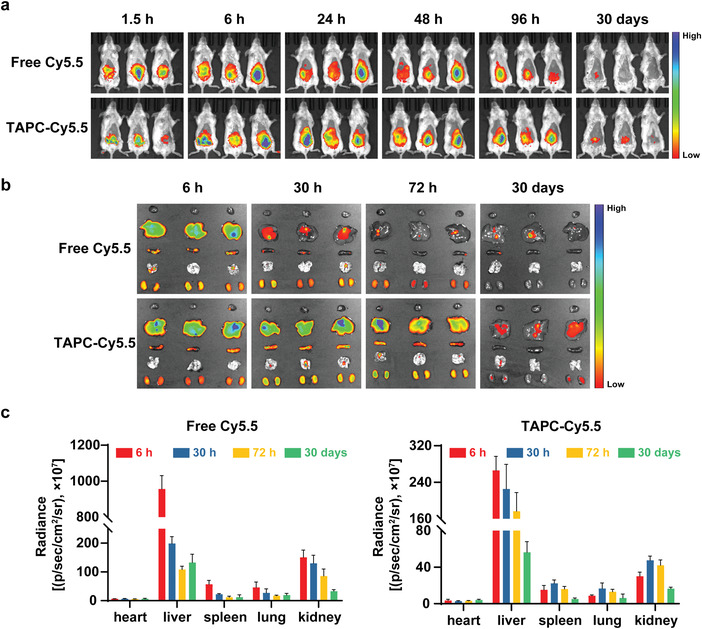
Distribution and excretion of TAPC‐4 in normal BALB/c mice via intraperitoneal injection. a) In vivo fluorescence imaging of the mice after injection of TAPC‐Cy5.5 or free Cy5.5 (*n* = 3). b) Ex vivo fluorescence imaging of heart, liver, spleen, lungs, and kidneys (*n* = 3). c) Quantification of the fluorescence intensity in the organs from panel (b). Cy5.5‐eq. dose, 0.1 mg kg^−1^ (Mean ± SEM).

Before evaluating the antineoplastic effect and safety of TAPC‐4 in vivo, we labeled TAPC‐4 with fluorescent Cy5.5 and studied its distribution and excretion via fluorescence imaging. BALB/c mice were separately treated with Cy5.5 and Cy5.5‐labeled TAPC‐4 (TAPC‐Cy5.5) via intraperitoneal injection. The mice were imaged in vivo, and their primary organs (heart, liver, spleen, lung, and kidney) were harvested for imaging ex vivo at different time intervals, including 1.5–96 h for the short term and 30 days for the long term. According to the in vivo fluorescence images (**Figure** **4**a), free Cy5.5 accumulation reached the maximum at 6 h, and most of it was rapidly excreted within 96 h. The fluorescence intensity of TAPC‐Cy5.5 came to the highest levels around 6 h, dropped down gradually, and got negligible after 30 days, indicating that TAPC‐4 can be excreted. Based on the fluorescence intensity in the harvested tissues, TAPC‐Cy5.5 and free Cy5.5 were mainly distributed in the liver, kidney, lung, and spleen. Compared with Cy5.5, which was rapidly cleared within 72 h, TAPC‐Cy5.5 was redistributed in the kidney, lung, and spleen within 30 h and slowly excreted, with only a small amount excreted at 72 h and most excreted after 30 days. It has been reported that water‐soluble fullerene derivatives accumulate in the liver, spleen, lung, and kidney and could be cleared from the body after a month,^[^ ^2^, ^27^ ^]^ which is consistent with the distribution and excretion behavior of TAPC‐4. Overall, TAPC‐4 is primarily distributed in the liver, spleen, lung, and kidney and gets excreted after several days, avoiding long‐term toxicity.

### 2.5 TAPC‐4 Inhibits Tumor Growth In Vivo

**Figure 5:**
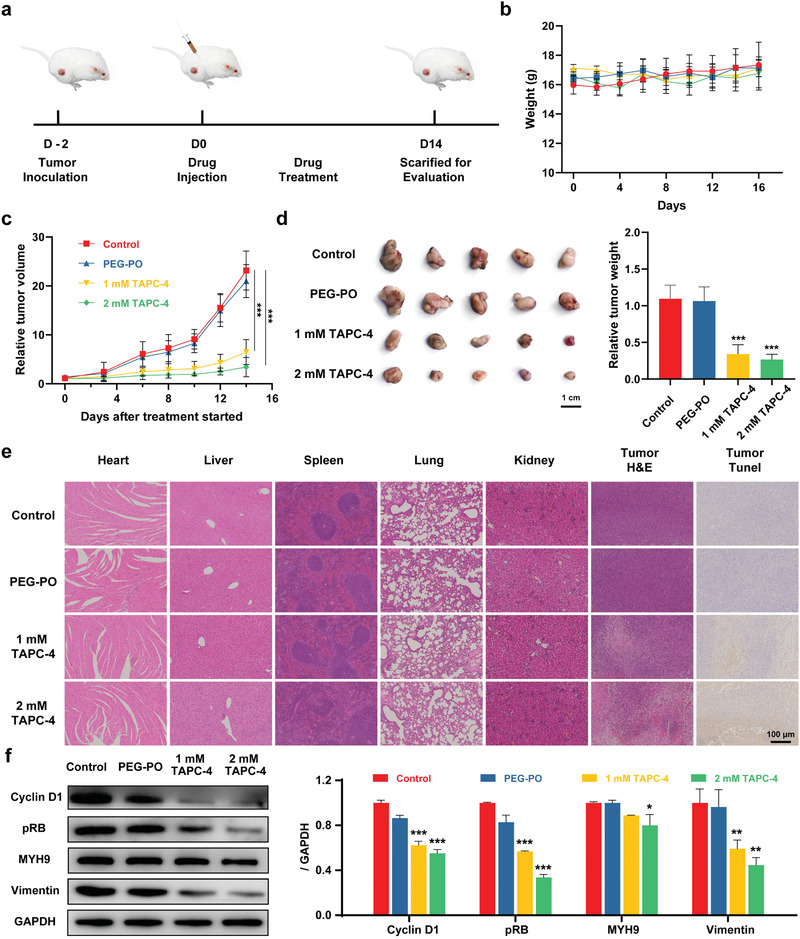
TAPC‐4 inhibits tumor growth in vivo. a) Scheme of 4T1 tumor model fabrication and treatments with saline, PEG‐PO, and PEG‐PO modified TAPC‐4 at different doses (*n* = 5). b) Bodyweight and c) tumor volume of the tumor‐bearing mice. d) Left: Photographs of tumor tissues collected on the 14th day. Right: Quantifications of tumor weight. e) H&E and TUNEL staining tissue sections of the heart, liver, spleen, lung, kidney, and tumor. The scale bar is 100 µm. f) Left: Protein levels of cyclin D1, pRB, MYH9, and vimentin in the tumor tissues. Right: Quantifications (Mean ± SEM; Student's *t*‐test, *p < 0.05, **p < 0.01, and ***p < 0.001).

This work is aimed to investigate the critical pharmacophore required for the antineoplastic effect of aminated fullerenes, providing theoretical strategies for the molecular design of aminated fullerenes in targeted tumor therapy. Accordingly, we only chose simple and common mouse tumor models to initially evaluate the in vivo antineoplastic effect and mechanism of TAPC‐4. Mice implanted subcutaneously with 4T1 cells were treated with 100 µL of saline, PEG‐PO, and PEG‐PO coated TAPC‐4 (low‐dose: 1 mm; high‐dose: 2 mm) via intraperitoneal injection at one‐day intervals, respectively (**Figure** **5**a). The body weight and tumor volume of the mice were monitored. No significant change in body weight and the weight of primary organs (heart, liver, spleen, lung, and kidney) was observed during the treatment, revealing the high biocompatibility of TAPC‐4 after PEG‐PO modification (Figure 5b; Figure S33, Supporting Information). As PEG‐PO did not cause changes in the tumor volume and tumor weight at a maximum dose, it gets negligible inhibitory effects on tumor growth (Figure 5c,d). TAPC‐4 shrunk the tumor tissues in a dose‐dependence, and an inhibition index of 75.5% based on tumor volume change was achieved at a high dose. TUNEL and H&E staining of tumor slices revealed that TAPC‐4 induced extensive tumor necrosis (Figure 5e). H&E‐stained slices of primary organs (heart, liver, spleen, lung, and kidney) showed no histological injury, indicating TAPC‐4 gets high safety at effective doses in vivo. The dramatically elevated number of white blood cells in tumor‐bearing mice dropped back to the normal range after TAPC‐4 treatments (Figure S34, Supporting Information).

In addition, TAPC‐4 reduced the protein level of cyclin D1 and thus abolished the phosphorylation of RB in tumor tissues, revealing that TAPC‐4 could induce G0/G1 cell cycle arrest to inhibit tumor proliferation in vivo. Besides, upregulation of E‐cadherin and downregulation of vimentin and N‐cadherin were revealed in the immunohistochemistry (IHC) analysis of tumor tissues (Figure S35, Supporting Information), and the vimentin expression in tumor tissues was also reduced by TAPC‐4 (Figure 5f), indicating the EMT process was reversed by TAPC‐4 in vivo. Therefore, TAPC‐4 of a wide safety margin gets notable antineoplastic effects on tumor growth via blocking the cell cycle in G0/G1 phase in vitro and in vivo. We will optimize the molecular structure of aminated fullerenes in the follow‐up work and choose specific tumors with overexpression of certain targets (e.g., HSP90*β*, vimentin, MYH9, cyclin D1) for antineoplastic efficacy and safety evaluation.

### 2.6 TAPC‐4 Inhibits Tumor Metastasis In Vivo

**Figure 6:**
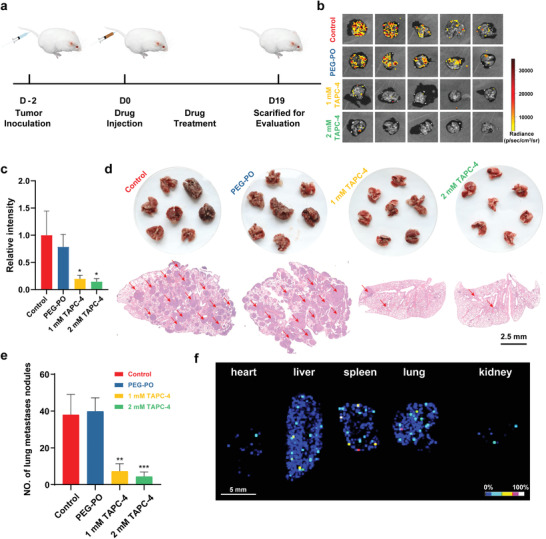
TAPC‐4 inhibits lung metastasis of melanoma in vivo. a) Scheme of lung metastatic melanoma model fabrication and treatments with saline, PEG‐PO, and PEG‐PO modified TAPC‐4 at different doses (*n* = 7). b) The lung luminescent images and c) relative luminescent intensity of B16‐F10‐Luc cells in the lung tissues (*n* = 5). d) Top: Macroscopic images of lung tissues. Bottom: H&E‐stained lung slices. The scale bar is 2.5 mm. e) Quantification of lung metastatic nodules (*n* = 7). f) MALDI‐TOF‐MS imaging of TAPC‐4 in primary organs. Mice were treated with 1 mm TAPC‐4 for 24 h, and the characteristic MS signal of C_60_ at 720 *m*/*z* was collected for the imaging (Mean ± SEM; Student's *t*‐test, *p < 0.05, **p < 0.01, and ***p < 0.001).

Lung metastatic melanoma was induced in BALB/c mice implanted intravenously with B16‐F10‐Luc cells. The mice were injected intravenously with 100 µL of saline, PEG‐PO, and PEG‐PO coated TAPC‐4 (low‐dose: 1 mm; high‐dose: 2 mm) at one‐day intervals (**Figure** **6**a). Tumor metastasis was quantitatively evaluated by the lung luminescent intensity and the number of metastatic nodules in lung tissues (Figure 6b–e). In the control groups, intense luminescent and numerous lung metastatic nodules were observed, indicating the metastatic rate was relatively high (Figure 6b–d). PEG‐PO with negligible antineoplastic activity did not change the metastatic rate. The luminescent intensity of melanoma dropped more than 80% when the mice received TAPC‐4 treatment, demonstrating an excellent therapeutic effect (Figure 6c). Correspondingly, the average number of lung metastatic nodules was reduced from 40 to 5 by TAPC‐4, indicating an inhibition index of 87.5% was achieved (Figure 6e). Meanwhile, TAPC‐4 distribution in the mice via intravenous injection was visualized by MALDI–TOF–MS imaging. As TAPC‐4 would dissociate into [60]fullerene under the excitation of the high‐energy MS laser, a specific MS signal of C_60_ (*m*/*z*: 720) was selected for the imaging. Consistent with the distribution behaviors revealed by fluorescence imaging (Figure 4b), TAPC‐4 was enriched in the lung, spleen, and liver after administration for 24 h (Figure 6f), and it did not cause any loss of mouse weight (Figure S36, Supporting Information), suggesting excellent biocompatibility of TAPC‐4. The distribution of TAPC‐4 in the lung, spleen, and liver could probably promote its inhibitory effect on the tumor metastasis to such tissues. Therefore, TAPC‐4 also exhibits a remarkable antineoplastic effect on tumor metastasis by reversing EMT in vitro and in vivo.

## 3 Conclusion

To explore the structure–activity relationship for antineoplastic aminated fullerenes, we have synthesized three derivatives of [60]fullerene with clarified amphiphilic molecular structures but the difference in the types and amounts of functional groups. TAPC‐4 can downregulate cyclin D1 expression and subsequently RB phosphorylation to block the cell cycle in G0/G1 phase, resulting in the inhibition of tumor cell proliferation with IC_50_ values of approximately 10 µm. TAPC‐3 with fewer amino moieties exhibits the same high antineoplastic effect. In contrast, TCPC‐4 with the same molecular configuration but the terminal amino groups replaced by carboxyl groups has a lower anti‐tumor effect. TAPC‐4 gets an inhibition index of 75.5% in tumor growth in vivo and causes no histological injury in primary organs, revealing the high antineoplastic efficacy and biosafety of TAPC‐4. Meanwhile, TAPC‐4 can abrogate the increased mobility and EMT of tumor cells and achieve an inhibition index of 87.5% in lung metastatic melanoma. In addition, tumor‐specific Hsp90*β*, vimentin, and MYH9 are screened as the targets of TAPC‐4. Molecular docking and MD simulation reveal that the amphiphilic molecular structure and the ending amino groups promote the binding of TAPC‐4 against Hsp90*β*, vimentin, and MYH9, probably resulting in the inhibition of cyclin D1 translation for the G0/G1 arrest, downregulation of vimentin expression for the EMT reversion, and subcellular transport of MYH9 for the cell mobility inhibition, respectively. Therefore, it is initially unveiled that the antineoplastic effect of aminated fullerenes is dominated by both the amphiphilic structure and the terminal amino moieties, providing fundamental insights and new strategies for their multi‐targeted anti‐tumor drug development.

## 4 Experimental Section

### Materials

C_60_ (purity: >99.5%) and cyclodextrin‐coated C_60_ were purchased from Beijing Fullcan Biotechnology Co., Ltd. 4‐N‐BOC‐aminopiperidine, 4‐piperidinecarboxylic acid tert‐butyl ester, and trifluoroacetic acid (TFA) were purchased from Innochem. Cumene hydroperoxide (CHP, 80%), d‐biotin *N*‐succinimidyl ester, Cy5.5 NHS ester, and dimethyl sulfide were purchased from Aladdin. PEG (*M*_w_ = 1900) and phosphorus oxychloride were purchased from Alfa Aesar. d‐luciferin potassium salt (luciferin) and cell counting kit‐8 (CCK‐8) were supplied by Yeasen. FxCycle PI/RNase staining solution was obtained from ThermoFisher. A549, DU145, U87, 4T1, B16‐F10, B16‐F10‐Luc, L02, and HUVEC cells were purchased from the Cell Resource Center of Peking Union Medical College.

### Characterizations

Purity of Boc‐protected TAPC‐4 and TAPC‐3 and tert‐butyl ester of TCPC was measured by high‐performance liquid chromatography (LC2030C 3D, Shimadzu) with parameters as follows: column (Agilent Eclipse XDB‐C18, 5 µm Silica 80 Å, 4.6 × 250 mm); mobile phase A (acetonitrile), mobile phase B (toluene), elution gradient (0 min 30% B, 20 min 70% B, 23 min 30% B, 30 min 30% B); flow rate (1 mL min^−1^); detection wavelength (310 nm); column temperature (30 °C); injection volume (10 µL). ESI‐MS was carried out with a mass spectrometer (Orbitrap Fusion LUMOs, Thermo Scientific). ^1^H and ^13^C NMR spectra were obtained by nuclear magnetic resonance spectroscopy (AVANCE 400 and AVANCE III 500WB, Bruker). Hydrochloride of TAPC‐4 and TAPC‐3 and sodium salt of TCPC‐4 were characterized by ultra‐performance liquid chromatography (I‐class Acquity, Waters) with parameters as follows: column (ACQUITY UPLC@BEH C18, 1.7 µm Silica 80 Å, 2.1 × 100 mm); mobile phase (water‐acetonitrile); column temperature (30 °C), flow rate (0.3 mL min^−1^). Mass spectrometry was further analyzed using a mass spectrometer (Orbitrap Fusion LUMOs, Thermo Scientific). Dynamic light scattering (DLS) and zeta potential were carried out on a NanoZS ZEN3600 (Malvern Instruments, Enigma Business Park, Britain). AFM was used to analyze size distribution (NanoWizard 4 NanoScience, JPK, Germany). Flow cytometry was performed with a flow cytometer (Attune NxT, Thermo Fischer, USA). Fluorescence imaging was performed with confocal microscopy (FV 1000‐IX81, Olympus, Japan). RT‐PCR analysis was carried out on a CFX96 Touch (Bio‐Rad, USA).

### Synthesis of Boc‐Protected TAPC‐4 and TAPC‐3

C_60_ (5 g) and 4‐N‐BOC‐aminopiperidine (13 g) were dissolved in chlorobenzene (625 mL), and cumene hydroperoxide (5 mL, 80%) was added. C_60_ was consumed after stirring (600 rpm) for 48 h at ambient temperature, as revealed by HPLC analysis. The reaction solution was stopped with dimethyl sulfide (10 mL), washed with saturated aqueous solutions of NH_4_Cl (300 mL) and NaHCO_3_ (300 mL), and dried with anhydrous Na_2_SO_4_. The crude product was obtained by evaporation at 60 °C as a reddish‐brown solid. Boc‐protected TAPC‐4 was purified by silica gel column chromatography (Rf: 0.3 with 25–30% ethyl acetate in toluene). As a by‐product of the reaction, Boc‐protected TAPC‐3 was obtained by silica gel column chromatography (Rf: 0.3 with 12–15% ethyl acetate in toluene).

### Synthesis of the Tert‐Butyl Ester of TCPC

C_60_ (5 g) and 4‐piperidinecarboxylic acid tert‐butyl ester (7.6 g) were dissolved in chlorobenzene (625 mL), and cumene hydroperoxide (5 mL) was added. C_60_ was consumed after stirring for 72 h at ambient temperature. The reaction solution was stopped with dimethyl sulfide and washed with the solution of NH_4_Cl and NaHCO_3_. Tert‐butyl ester of TCPC was obtained after evaporation and purification with silica gel column chromatography (Rf: 0.3 with 20% ethyl acetate in toluene).

### Synthesis of TAPC‐4, TAPC‐3, and TCPC‐4

Deprotection of Boc groups was carried out in 10% TFA/CHCl_3_ (10%) for 6 h at RT, and trifluoroacetic acid salts of TAPC‐4 and TAPC‐3 were obtained via rotary evaporation. The hydrochloride salt of TAPC‐4 and TAPC‐3 was obtained by exchanging trifluoroacetic acid ions with chloride ions and further purified by dialysis against ultrapure water for 48 h. The sodium salt of TCPC‐4 was obtained by removing tert‐butyl groups in 50% TFA/CHCl_3_ (10%) for 2 h at RT, dried, and dissolved in aqueous solutions of NaOH (100 µm), and dialyzed against ultrapure water for 48 h.

### Modification of TAPC‐4 and TAPC‐3 with PEG‐PO

PEG‐PO was prepared with PEG (*M*_w_ = 1900) and phosphorus oxychloride according to the reported method.^[^ ^10^ ^]^ TAPC‐3 and TAPC‐4 were separately mixed with a tenfold molar ratio of PEG‐PO in an aqueous solution for further in vivo and in vitro experiments.

### Labeling of TAPC‐4 with d‐Biotin

d‐biotin‐NHS in DMF (5 mm) was mixed with the aqueous solution of TAPC‐4 (1 mm), and the mixture was stirred for 1 h under dark. Excessive DMSO and d‐Biotin‐NHS were removed by dialysis against ultrapure water and purification with a Sephadex G‐25 column. A mixture of biotin‐labeled TAPC‐4 and residual TAPC‐4 was obtained as revealed by LC‐ESI/MS.

### Labeling of TAPC‐4 with Cy5.5

Cy5.5‐NHS ester in DMF was added into the aqueous solution of TAPC‐4 in a 1:5 molar ratio of Cy5.5‐NHS to TAPC‐4. The reaction was stirred at room temperature and under dark for 3 h, and the residual DMF was removed by dialysis to obtain TAPC‐Cy5.5.

### Optimization of Molecular Structure By Theoretical Calculations

Molecular structures of TAPC‐4, TCPC‐4, Boc‐protected TAPC‐3, and biotin‐labeled TAPC‐4 were first optimized with original pm3, and b3lyp/3‐21g* to speed up the computational process, and the final optimization and the frontier MOs were carried out by b3lyp/6‐31g*. The above calculations were done using the Gaussian 09 quantum chemical program package.

### Cytotoxicity In Vitro

The half inhibitory concentrations (IC_50_) of TAPC‐3, TAPC‐4, TCPC‐4, and cyclodextrin‐coated C_60_ were evaluated in A549, DU145, U87, 4T1, B16‐F10, B16‐F10‐Luc, L02, and HUVEC cells. Cells were cultured in 96‐well plates for 24 h and incubated with TAPC‐3 and TACP‐4 at concentrations from 0 to 20 µm and TCPC‐4 at concentrations from 0 to 200 µm, and cyclodextrin‐coated C_60_ at concentrations from 0 to 100 µm in the dark at 37 °C for 24 h, respectively. Cell viability was detected by cell counting kit‐8 (CCK‐8; DOJINDO, Kumamoto, Japan). The absorption value of CCK‐8 at 450 nm was read with a 96‐well plate reader (iMark microplate reader, Bio‐RAD, USA) to determine the viability (cell viability = (OD_tre_ − OD_medium_) / (OD_con_ − OD_medium_)), where OD_tre_ is the absorption value at 450 nm of treated cells, OD_con_ is that of control cells, and OD_medium_ is that of the culture medium.

### Proteomic Analysis

A549 cells treated with TAPC‐4 (10 µm) for 24 h were lysed with RIPA to extract proteins. Proteins were purified by sixfold acetone precipitation overnight at −20 °C and re‐suspended for concentration estimation by BCA assay. Proteins of the treating group and control group were prepared in triplicate. Every 100 µg protein was digested into peptides with 2.5 µg trypsin overnight at 37 °C. The peptides were labeled with iTRAQ Reagent‐8plex (AB Sciex iTRAQ, catalog No. 4390812) at room temperature for 2 h. The peptide solutions were lyophilized and followed by resuspension in 2% acetonitrile and 98% water (pH 10) for fractionation. Isotope labeling samples were fractionated on an Ultimate 3000 HPLC Dionex NCS‐3500 system with an RP C18 column at 35 °C. All fractions were analyzed on an Ultimate 3000 HPLC nanoViper FS system coupled to a Q‐Exactive instrument (Thermo Fisher Scientific) with a nanoelectrospray source. The analysis was performed with a 25‐cm column at 35 °C and a 65‐min gradient at a flow rate of 300 nL min^−1^ ramping from 97% buffer A (99.9% water and 0.1% formic acid) to 30% buffer B (99.9% acetonitrile and 0.1% formic acid) in 48 min, to 80% B in 1 min, kept 5 min, to 3% B in 0.5 min and kept 10.5 min. The mass spectrometers were operated in DDA mode to automatically isolate and fragment the top 25 multiply charged precursors according to their intensities. The MS1 scanning mode ranged from 350 to 1600 *m*/*z*, with a resolution of 70 000, AGC 3e6, and a maximum injection time of 50 ms. The MS2 scanning of fragment ions was set to 17 500 resolution, AGC 2e5, maximum injection time 100 ms, dynamic exclusion 18.0 s, isolation window 2.0 *m*/*z*, and NCE 29. The MS data were searched in the *homo sapien* (human) protein database using the proteome discoverer software (PD software) with the SEQUEST algorithm computational analysis. CapitalBio Technology carried out the proteomic analysis.

### Cell Migration Assays

Cell migration was measured using transwell chambers. Cells were suspended in a serum‐free medium and added to the upper chamber. The lower chamber was filled with medium containing 20% FBS. After incubation for 6 h, the number of cells passing through the lower surface of the insert was dyed and counted.

### RNA Isolation and RT‐PCR

The total RNA of the cultured cells was extracted using TRIzol S3 (Invitrogen Life Technologies, 15596‐026) according to the manufacturer's protocol. Total RNA (1 µg) was converted to cDNA using 1st Strand cDNA Synthesis SuperMix (NovoScript, E044‐01A) following the manufacturer's instructions. RT‐PCR was performed in a 20‐µL reaction volume according to the manufacturer's protocol using SYBR One‐Step qRT‐PCR Kit (NovoScript, E092‐01A).

### Western Blot

Proteins were extracted in lysis buffer containing protease inhibitor cocktail and phosphatase inhibitor. The collected proteins were boiled in a 5× loading buffer for 5 min, resolved by SDS–polyacrylamide gels, and then transferred to the PVDF membrane. After blocking by 5% skimmed milk at 25 °C for 1 h, membranes were incubated with primary antibodies at 4 °C overnight and then incubated with secondary antibodies at 25 °C for 1 h. At last, protein bands were visualized using an enhanced chemiluminescence assay (ECL, Absin, abs920).

### Immunofluorescence Imaging

Cells after treatments were fixed with methanol for 5 min at room temperature, blocked in PBS containing 3% BSA for 1 h at room temperature, and incubated with primary antibodies overnight at 4 °C. Alexa Fluor conjugated secondary antibodies were incubated for 1 h at room temperature. Hoechst 33342 was then used to stain nuclei, and fluorescence images were captured by laser scanning confocal microscopy.

### Antibodies

Antibodies were listed as follows: Phospho‐Rb (Ser807/811) (D20B12) XP Rabbit mAb (Cell Signaling Technology, Cat: 8516), Cyclin D1 (E3P5S) XP Rabbit mAb (Cell Signaling Technology, Cat: 55506), GAPDH (D16H11) XP Rabbit mAb (Cell Signaling Technology, Cat: 5174), Vimentin Polyclonal Antibody (Proteintech, Cat: 10366‐1‐AP), N‐Cadherin Polyclonal Antibody (Proteintech, Cat: 22018‐1‐AP), Anti‐non‐muscle Myosin IIA antibody [EPR22933‐9] (Abcam, Cat: ab238131), Anti‐MYH9 Polyclonal Antibody (Beijing Solarbio Science & Technology Co., Ltd.), Anti‐Hsp90 antibody [EPR16621‐67] (Abcam, Cat: ab203126), Anti‐E Cadherin antibody [SP64] (Abcam, Cat: ab227639).

### Binding Target Identification for TAPC‐4

Briefly, biotin‐labeled TAPC‐4 (≈10 nmol) and A549 cell lysate (1 mg) were incubated overnight at 4 °C. The complexes of biotin‐labeled TAPC‐4 and binding proteins were enriched by streptavidin‐modified beads after incubation at 4 °C for 1 h. The collected beads were washed three times with 1× PBS containing 0.1% NP‐40. The bound proteins were eluted via heating in a loading buffer (30 µL), separated by 10% polyacrylamide gel electrophoresis, and stained with Coomassie blue. Specific protein bands were excised, digested, and identified by LC‐MS at Capital Bio Beijing (Beijing, China), and the proteins in bands 1–5 were listed in Table S1, Supporting Information.

### Molecular Docking

Molecular docking simulation was carried out using the Autodock version 4.2.6.20 software. The crystal structure of protein Hsp90‐beta (PDB code 5FWK) obtained from the RCSB PDB databank was set as the initial structure. 3D protein structures of MYH9 and vimentin were extracted from the AlphaFold modeling protein structure database. In the molecular docking process, each protein was set as the receptor, and TAPC‐4 or TCPC‐4 was set as the ligand. Meanwhile, the AutoDockTools version 1.5.620 module was used to generate docking input files and analyze docking results. The grid box size was set as 60 × 60 × 60 points with the spacing between grid points at 0.375Å, which covered almost the whole binding site for each protein. Binding affinity map calculation was conducted by the software AutoGrid. For each docking process, one total of 100 docking trials with at least 150 docking conformations sizes were executed with random starting positions for receptor and ligand. The final docked conformations were clustered using a 1.5 Å root mean square deviation (RMSD) tolerance. The binding pose with the lowest docking energy from the most significant cluster was chosen for further analysis.

### Molecular Dynamics Simulations

Each initial model of the docking conformation was modeled with the XleaP module, and the ligand and protein were parameterized by adding gaff and ff14SB force fields. The complex system was neutralized with sodium ions and dissolved in a TIP3P water cubic box with a solvent layer of 10 Å from the box edge to the solute. MD simulations were then performed to relax structures. Each complex was gradually heated from 0 to 300 K within 100 ps and then equilibrated for 100 ps at 300 K using a combination of NVT (constant composition, volume, and temperature). The system's temperature, energy and density, and the backbone's root mean square deviation (RMSD) convergence were used to monitor the equilibrium. The binding free energy (∆Gbind) of each protein‐ligand system was computed for each snapshot and averaged using the MMGBSA module in Amber14 software. Finally, at least 1000 snapshots from the >10 ns MD simulation trajectory were used to extract the final average structure of the complex.

### Animal Experiments

Animal models were constructed with six‐week‐old female Balb/c mice purchased from the Perking University Laboratory Animal Center, China. All the animal experiments were conducted according to protocols approved by the Institutional Animal Care and Use Committee in the Institute of Chemistry, Chinese Academy of Sciences (approval number is SYXK (Jing) 2018‐0033). Mice were housed under controlled conditions of ≈50% humidity, ≈25 °C temperature, and a 12 h light/dark cycle. Isoflurane was used for anesthesia. At the end of the experiments, mice were euthanized under CO_2_ anesthesia.

### In Vivo Real‐Time Fluorescence Imaging

BALB/c mice were randomly divided into two groups (*n* = 3). Hair on the abdominal side of the mice was removed by depilatory cream to reduce hair‐derived autofluorescence. TAPC‐Cy5.5 and free Cy5.5 were given to the mice by intraperitoneal injection at a 100 µg kg^−1^ Cy5.5‐eq. dose. After the injection for 1.5 h, 6 h, 24 h, 48, 96 h, and 30 days, the mice were anesthetized and then imaged by the IVIS Spectrum system (PerkinElmer, USA).

### Ex Vivo Fluorescence Imaging

Twenty‐four BALB/c mice were randomly divided into two groups. TAPC‐Cy5.5 and free Cy5.5 were given to the mice by intraperitoneal injection at a 100 µg kg^−1^ Cy5.5‐eq. dose. After the injection for 6 h, 24 h, 48, 96 h, and 30 days, every three mice were sacrificed by cervical dislocation, and the primary organs (heart, liver, spleen, lungs, kidneys) were harvested and imaged by the IVIS Spectrum system. The fluorescence intensities of the organs were quantified.

### TAPC‐4 Inhibiting Tumor Growth In Vivo

Female BALB/c mice were subcutaneously injected with 1 × 10^7^ 4T1 cells. The 4T1 tumor‐bearing mice were randomly grouped (five mice per group) and separately treated with 100 µL of saline, PEG‐PO (20 mm), and PEG‐PO coated TAPC‐4 (low‐dose: 1 mm; high‐dose: 2 mm) via intraperitoneal injection (a total of seven times) at a one‐day interval from the 3rd to 14th day after tumor injection. Subcutaneous tumor sizes were monitored every 2 days for 2 weeks by a digital caliper. The tumor volume was calculated as the following formula: tumor volume (mm^3^) = 1/2 × (length × width × width).

### TAPC‐4 Inhibiting Tumor Metastasis In Vivo

BALB/c mice were intravenously injected with 1 × 10^7^ B16‐F10‐Luc cells to build the mouse model of lung metastasis of malignant melanoma. The mice were randomly grouped (seven mice per group) and separately treated with 100 µL of saline, PEG‐PO (20 mm), PEG‐PO coated TAPC‐4 (low‐dose: 1 mm; high‐dose: 2 mm) via tail vein injection (a total of ten times) at a one‐day interval since the 3rd day after tumor injection. On the 19th day, tumor volumes were evaluated by bioluminescence intensity of B16‐F10‐Luc cells with IVIS Spectrum (PerkinElmer). Intraperitoneal injection of D‐luciferin solution (30 mg mL^−1^, 100 µL) was performed 20 min before imaging. Tumors that metastasized to the lungs were also observed by H&E staining.

### Matrix‐Assisted Laser Desorption Ionization Time of Flight Mass Spectrometry (MALDI‐TOF‐MS) Imaging of TAPC‐4 In Vivo

Frozen tissues (heart, liver, spleen, lung, kidney) were sectioned in 12 µm thickness at −18 °C with a Leica CM1950 cryostat (Leica Microsystems GmbH, Wetzlar, Germany) and mounted on indium tin oxide (ITO) coated glass slides (Bruker Daltonics, Bremen, Germany). MALDI‐TOF‐MS imaging was performed in positive ion mode using a mass spectrometer with a SmartBeam laser (355 nm, Ultraflextreme, Bruker Daltonics, Bremen, Germany). Mass calibration was performed using the standard external method. The analyzer was operated in positive reflection mode, and each spectrum was acquired as the average of 500 laser shots at a frequency of 200 Hz. All data were processed with FlexAnalysis (Bruker Daltonics) and FlexImaging (Bruker Daltonics).

### Histopathological Analysis

Collected tissues (heart, spleen, kidneys, liver, lung, and tumor) were fixed in formalin, embedded in paraffin wax blocks, sliced, deparaffinized in xylene, and rehydrated through an alcohol gradient. For H&E staining, the slides were stained with hematoxylin and eosin. For oil red O staining, fixed tissues by formalin were treated in sucrose solution (15% and 30%) overnight and inserted in an optical cutting temperature compound (OCT). Slices of 6–8 µm were prepared using a freezing microtome and dealt with standard methods, including washing, staining, differentiation, and sealing. For immunohistochemistry staining, tissue sections of the tumor were stained with corresponding antibodies following the manufacturer's protocols. The slices were scanned by the scanning microtome (KF‐PRO‐005, KFBIO, China) and analyzed with ImageJ 7.0 software.

### Statistical Analysis

Statistical significance between treatment groups was determined via a two‐tailed Student's *t*‐test, assuming unequal variances. Comparisons between multiple groups were determined using a one‐way analysis of variance (ANOVA) with Tukey's post hoc test. Calculations were performed using GraphPad Prism software. *p*‐values less than 0.05 were considered significant.

## Conflict of Interest

The authors declare no conflict of interest.

## Supporting information

Supporting Information

## Data Availability

The data that support the findings of this study are available in the supplementary material of this article.
