# Supplementary material for: Amphiphilic Aminated Derivatives of [60]Fullerene as Potent Inhibitors of Tumor Growth and Metastasis
Source: Adv Sci (Weinh). 2022 Aug 28;9(29):2201541. doi: 10.1002/advs.202201541 (PMC9561876; doi:10.1002/advs.202201541)
Supplement: Supplementary file 1 — Supporting Information [file ADVS-9-2201541-s001.pdf]

Supporting Information

Supporting Information is available from the Wiley Online Library or from the author.

Acknowledgments

This work is supported by the major research project of the National Natural Science Foundation of China (51802310; 92061123; 52172055) and the Key Research Program of the Chinese Academy of Sciences (QYZDJ-SSW-SLH025).

References

[1] D. Hanahan, Robert, *Cell* **2011**, *144* (5), 646.

[2] a) C. Chen, G. Xing, J. Wang, Y. Zhao, B. Li, J. Tang, G. Jia, T. Wang, J. Sun, L. Xing, H. Yuan, Y. Gao, H. Meng, Z. Chen, F. Zhao, Z. Chai, X. Fang, *Nano Lett.* **2005**, *5* (10), 2050; b) Y. Liu, C. Chen, P. Qian, X. Lu, B. Sun, X. Zhang, L. Wang, X. Gao, H. Li, Z. Chen, J. Tang, W. Zhang, J. Dong, R. Bai, P. E. Lobie, Q. Wu, S. Liu, H. Zhang, F. Zhao, M. S. Wicha, T. Zhu, Y. Zhao, *Nat. Commun.* **2015**, *6*, 5988; c) M. Zhen, C. Shu, J. Li, G. Zhang, T. Wang, Y. Luo, T. Zou, R. Deng, F. Fang, H. Lei, C. Wang, C. Bai, *Science China Materials* **2015**, *58* (10), 799; d) L. Li, M. Zhen, H. Wang, Z. Sun, W. Jia, Z. Zhao, C. Zhou, S. Liu, C. Wang, C. Bai, *Nano Lett.* **2020**, *20* (6), 4487.

[3] a) S.-G. Kang, G. Zhou, P. Yang, Y. Liu, B. Sun, T. Huynh, H. Meng, L. Zhao, G. Xing, C. Chen, Y. Zhao, R. Zhou, *Proceedings of the National Academy of Sciences* **2012**, *109* (38), 15431; b) Y. Pan, L. Wang, S.-G. Kang, Y. Lu, Z. Yang, T. Huynh, C. Chen, R. Zhou, M. Guo, Y. Zhao, *ACS Nano* **2015**, *9* (7), 6826; c) J. Liu, S.-g. Kang, P. Wang, Y. Wang, X. Lv, Y. Liu, F. Wang, Z. Gu, Z. Yang, J. K. Weber, N. Tao, Z. Qin, Q. Miao, C. Chen, R. Zhou, Y. Zhao, *Biomaterials* **2018**, *152*, 24; d) K. S. Siddiqi, A. Husen, R. A. K. Rao, *J. Nanobiotechnol.* **2018**, *16* (1).

[4] Z. Wang, Z. Lu, Y. Zhao, X. Gao, *Nanoscale* **2015**, *7* (7), 2914.

[5] a) T. Yasuno, T. Ohe, H. Ikeda, K. Takahashi, S. Nakamura, T. Mashino, *Int. J. Nanomedicine* **2019**, *14*, 6325; b) C.-W. Wong, A. V. Zhilenkov, O. A. Kraevaya, D. V. Mischenko, P. A. Troshin, S.-h. Hsu, *J. Med. Chem.* **2019**, *62* (15), 7111.

[6] W. Zhou, J. Huo, Y. Yang, X. Zhang, S. Li, C. Zhao, H. Ma, Y. Liu, J. Liu, J. Li, M. Zhen, J. Li, X. Fang, C. Wang, *ACS Appl. Mater. Interfaces* **2020**, *12* (51), 56862.

[7] X. Zhang, W. Zhou, Y. Liu, L. Jin, J. Huo, Y. Yang, S. Li, H. Ma, J. Li, M. Zhen, J. Li, C. Wang, *Nano Research* **2021**.

[8] L. Lemiègre, T. Tanaka, T. Nanao, H. Isobe, E. Nakamura, *Chem. Lett.* **2007**, *36* (1), 20.

[9] M. Chen, S. Zhou, L. Guo, L. Wang, F. Yao, Y. Hu, H. Li, J. Hao, *Langmuir*

2019, *35* (21), 6939.
[10]H. B. Na, I. S. Lee, H. Seo, Y. I. Park, J. H. Lee, S. W. Kim, T. Hyeon, *Chem. Commun. (Camb.)* **2007**, (48), 5167.
[11]C. Giacinti, A. Giordano, *Oncogene* **2006**, *25* (38), 5220.
[12]a) C. Y. Jiao, Q. C. Feng, C. X. Li, D. Wang, S. Han, Y. D. Zhang, W. J. Jiang, J. Chang, X. Wang, X. C. Li, *Cell Death Dis.* **2021**, *12* (1); b) H. B. Low, Y. Zhang, *Immune Network* **2016**, *16* (2), 85; c) X. Wang, L. Yin, L. Yang, Y. Zheng, S. Liu, J. Yang, H. Cui, H. Wang, *The FEBS Journal* **2019**, *286* (24), 4889; d) S. Zhang, Y. Wang, S. Chen, J. Li, *Biomed Pharmacother* **2018**, *106*, 1396.
[13]S. Usman, N. H. Waseem, T. K. N. Nguyen, S. Mohsin, A. Jamal, M.-T. Teh, A. Waseem, *Cancers* **2021**, *13* (19), 4985.
[14]Y. Wang, J. Shi, K. Chai, X. Ying, B. Zhou, *Curr. Cancer Drug Targets* **2013**, *13* (9), 963.
[15]S. Wu, Y. Du, J. Beckford, H. Alachkar, *J. Transl. Med.* **2018**, *16* (1).
[16]C. Chen, L. Yin, X. Song, H. Yang, X. Ren, X. Gong, F. Wang, L. Yang, *Biochem. Biophys. Res. Commun.* **2016**, *469* (1), 132.
[17]H. Peinado, D. Olmeda, A. Cano, *Nat. Rev. Cancer* **2007**, *7* (6), 415.
[18]C.-Y. Loh, J. Chai, T. Tang, W. Wong, G. Sethi, M. Shanmugam, P. Chong, C. Looi, *Cells* **2019**, *8* (10), 1118.
[19]S. Yin, V. T. Cheryan, L. Xu, A. K. Rishi, K. B. Reddy, *PLoS ONE* **2017**, *12* (8), e0183578.
[20]K. Strouhalova, M. Přechová, A. Gandalovičová, J. Brábek, M. Gregor, D. Rosel, *Cancers* **2020**, *12* (1), 184.
[21]A. Cano, M. A. Pérez-Moreno, I. Rodrigo, A. Locascio, M. J. Blanco, M. G. Del Barrio, F. Portillo, M. A. Nieto, *Nat. Cell Biol.* **2000**, *2* (2), 76.
[22]a) S. Chatterjee, T. Burns, *Int. J. Mol. Sci.* **2017**, *18* (9), 1978; b) J. Wu, T. Liu, Z. Rios, Q. Mei, X. Lin, S. Cao, *Trends Pharmacol. Sci.* **2017**, *38* (3), 226.
[23]S. K. Wandinger, K. Richter, J. Buchner, *J. Biol. Chem.* **2008**, *283* (27), 18473.
[24]a) A. D. Basso, D. B. Solit, G. Chiosis, B. Giri, P. Tsichlis, N. Rosen, *J. Biol. Chem.* **2002**, *277* (42), 39858; b) R. C. Muise-Helmericks, H. L. Grimes, A. Bellacosa, S. E. Malstrom, P. N. Tsichlis, N. Rosen, *J. Biol. Chem.* **1998**, *273* (45), 29864.
[25]a) G. Lahat, Q.-S. Zhu, K.-L. Huang, S. Wang, S. Bolshakov, J. Liu, K. Torres, R. R. Langley, A. J. Lazar, M. C. Hung, D. Lev, *PLoS ONE* **2010**, *5* (4), e10105; b) A. K. Kanugula, V. M. Dhople, U. Völker, R. Ummanni, S. Kotamraju, *PLoS ONE* **2014**, *9* (9), e108890.
[26]a) A. Pecci, X. Ma, A. Savoia, R. S. Adelstein, *Gene* **2018**, *664*, 152; b) M. T. Breckenridge, N. G. Dulyaninova, T. T. Egelhoff, *Mol. Biol. Cell* **2009**, *20* (1), 338.
[27]Y. Zhou, R. Deng, M. Zhen, J. Li, M. Guan, W. Jia, X. Li, Y. Zhang, T. Yu, T. Zou, Z. Lu, J. Guo, L. Sun, C. Shu, C. Wang, *Biomaterials* **2017**, *133*, 107.

TAPC-4, an amphiphilic amino fullerene derivative with a well-defined structure, prevents tumor growth and metastasis by blocking the cell cycle in G0/G1 phase, reversing the EMT process and regulating the localization of MYH9 protein, which may be achieved by binding Hsp90-beta, Vimentin, and MYH9 proteins, respectively, and the certainty of the molecular structure provides novel ideas to investigate the potential targets of fullerene biological effects.

Keywords: amphipathic aminated [60]fullerene, cell cycle arrest, cell mobility suppression, mesenchymal-epithelial transition, protein target

Jiawei Huo, Jie Li*, Yang Liu, Libin Yang, Xinran Cao, Chong Zhao, Yicheng Lu, Wei Zhou, Shumu Li, Jianan Liu, Jiao Li, Xing Li, Jing Wan, Rui Wen, Mingming Zhen, Chunru Wang*, Chunli Bai*

Amphiphilic Aminated Derivatives of [60]Fullerene as Potent Inhibitors of Tumor Growth and Metastasis

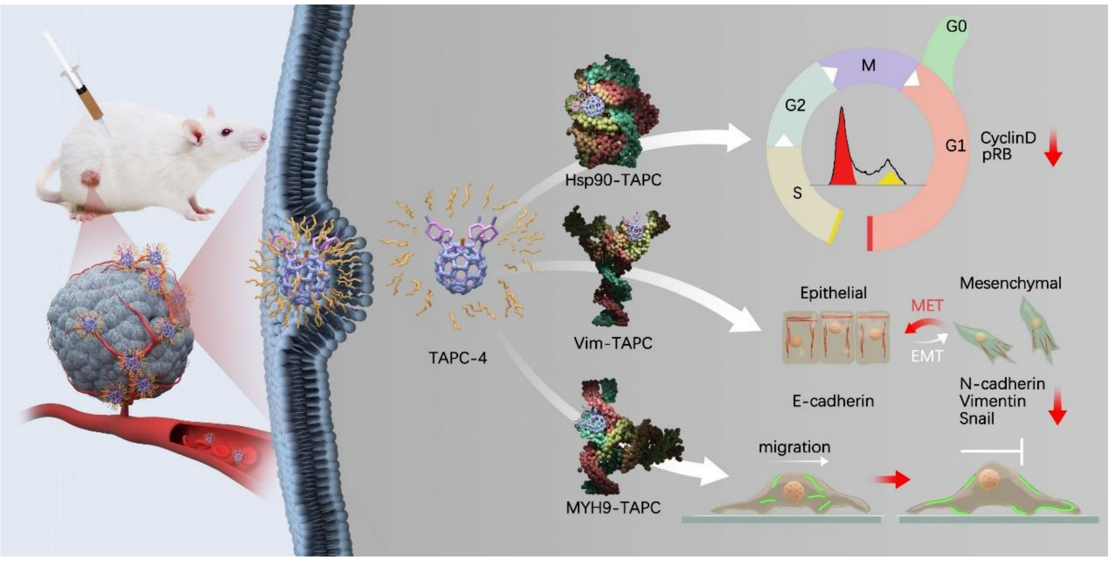

Supporting Information

Amphiphilic Aminated Derivatives of [60]Fullerene as Potent Inhibitors of Tumor Growth and Metastasis

Jiawei Huo, Jie Li, Yang Liu, Libin Yang, Xinran Cao, Chong Zhao, Yicheng Lu, Wei Zhou, Shumu Li, Jianan Liu, Jiao Li, Xing Li, Jing Wan, Rui Wen, Mingming Zhen, Chunru Wang*, Chunli Bai**

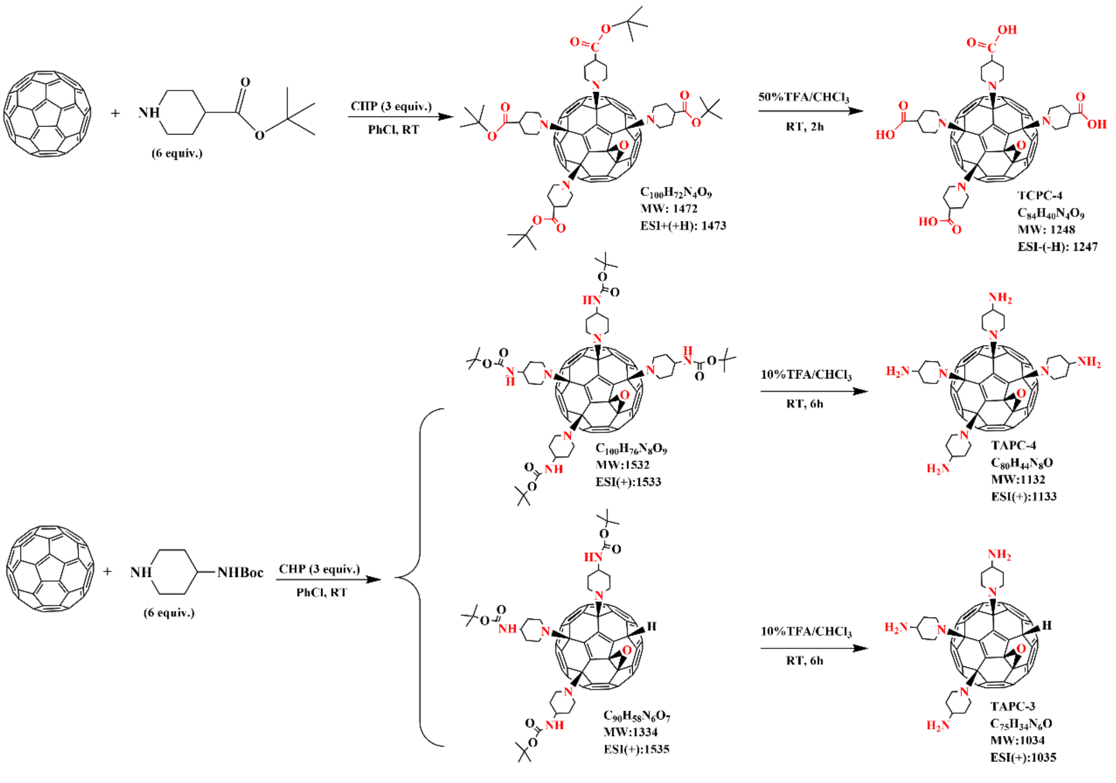

Figure S1: Schematic synthesis of TAPC-3, TAPC-4, and TCPC-4.

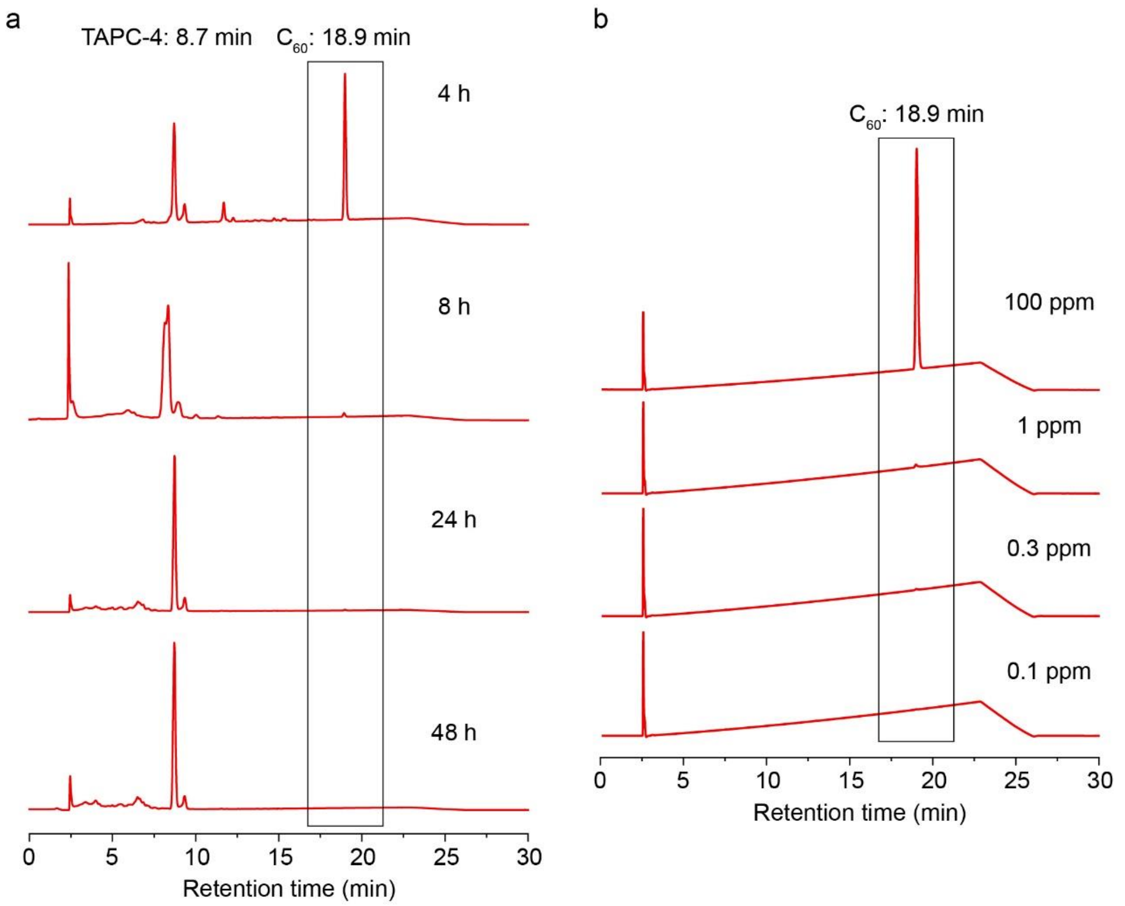

Figure S2: (a) The progression of the TAPC-4 synthesis reaction was detected at 4 h, 8 h, 24 h, and 48 h. (b) Limit of detection of C_{60}.

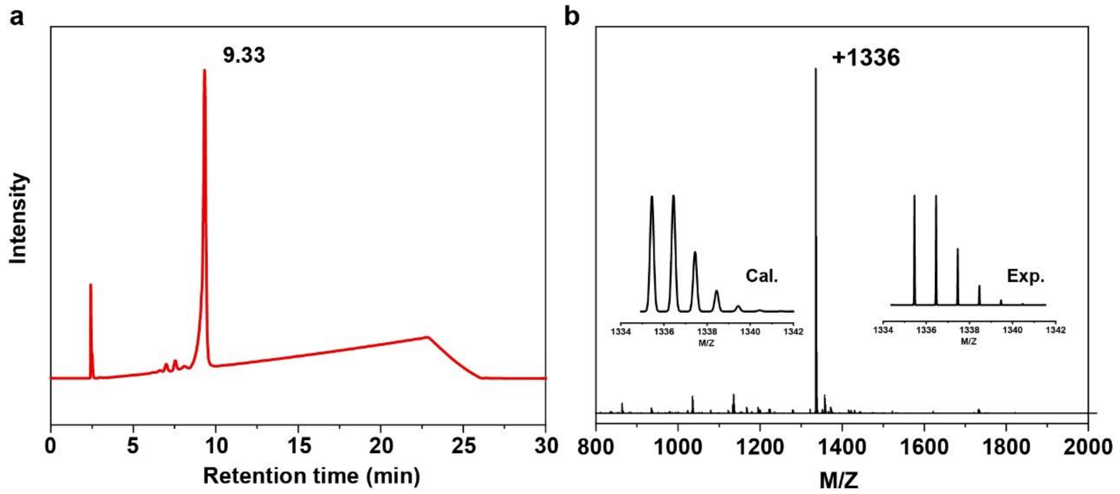

Figure S3: (a) HPLC analysis of Boc-protected TAPC-3. (b) ESI mass spectra of Boc-protected TAPC-3. Insets show the calculated and experimental isotope distribution patterns, respectively.

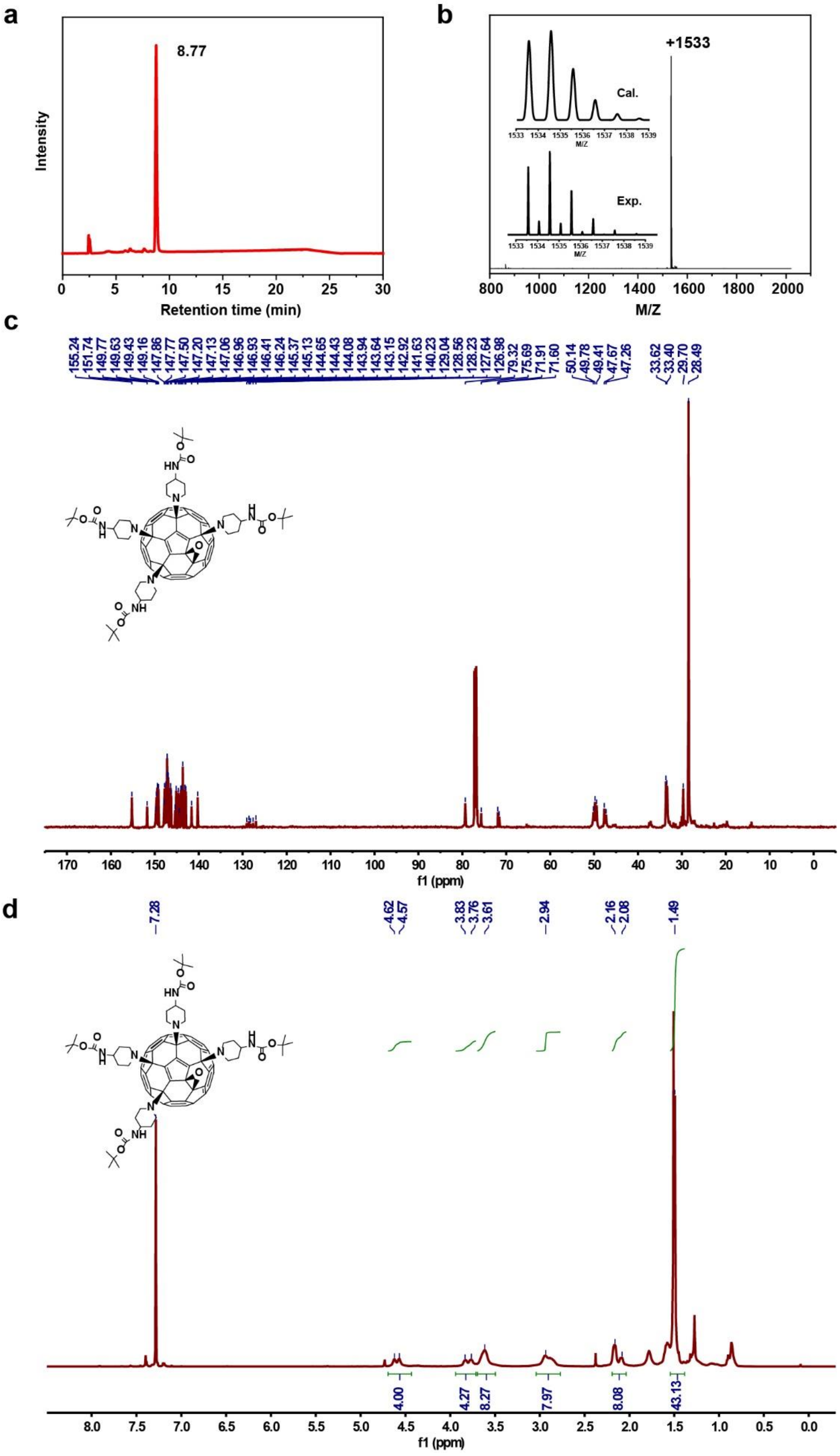

Figure S4: (a) HPLC analysis of Boc-protected TAPC-4. (b) ESI mass spectra of Boc-protected TAPC-4. Insets show the calculated and experimental isotope distribution patterns, respectively. (c)

^{13}C-NMR and (d) ^{1}H-NMR spectrum of Boc-protected TAPC-4.

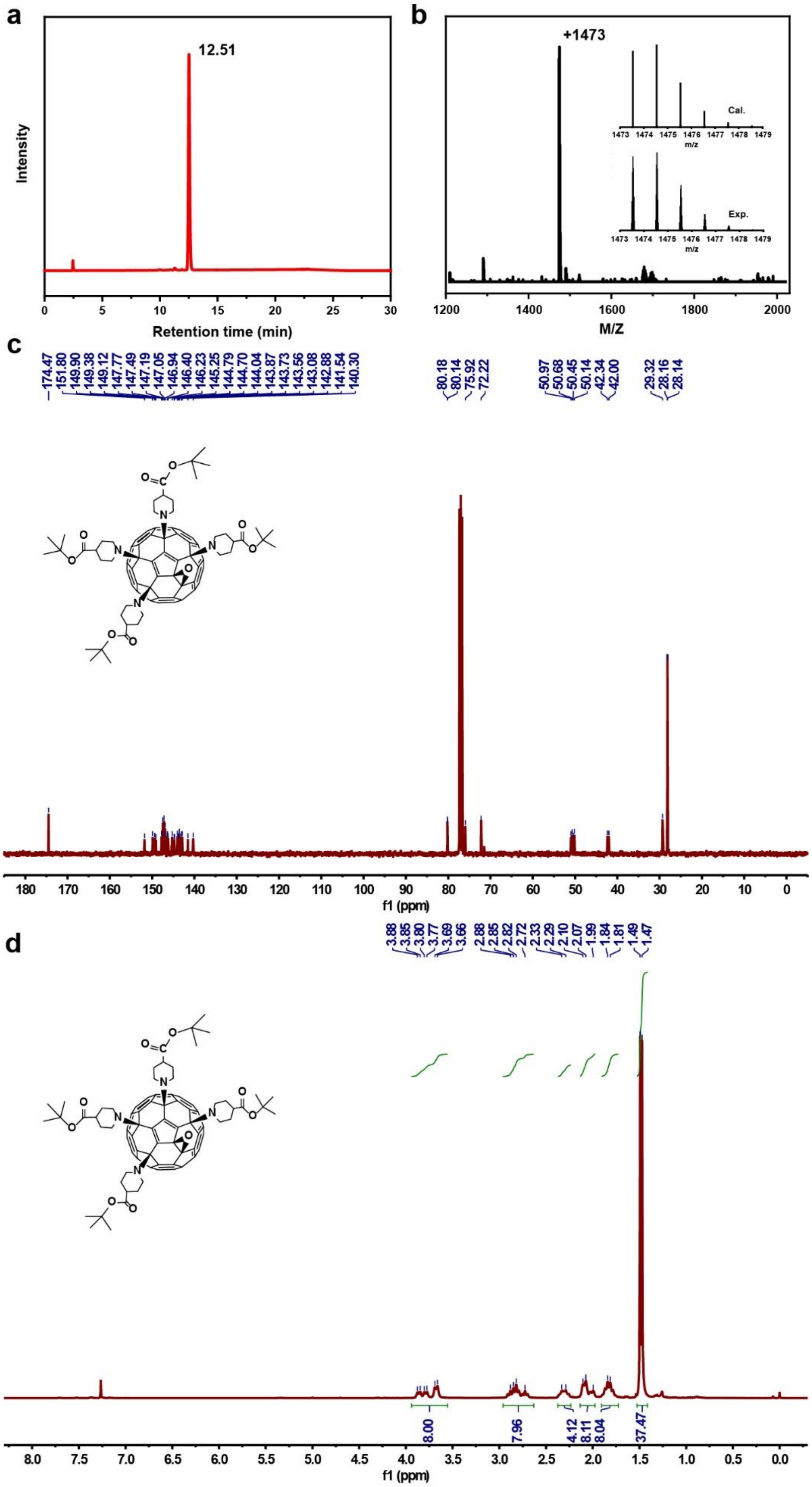

Figure S5: (a) HPLC analysis of tert-butyl ester of TCPC-4. (b) ESI mass spectra of the tert-butyl

ester of TCPC-4. Insets show the calculated and experimental isotope distribution patterns, respectively. (c) ^{13}C-NMR and (d) ^{1}H-NMR spectrum of the tert-butyl ester of TCPC-4.

Table S1: Energy calculation for different isomers of Boc-protected TAPC-3.

Name	Relative HF (kcal/mol)	LUMO-HOMO (kcal/mol)
TAPC3-Boc-2	0	68.23
TAPC3-Boc-1	-5.849141551	68.25

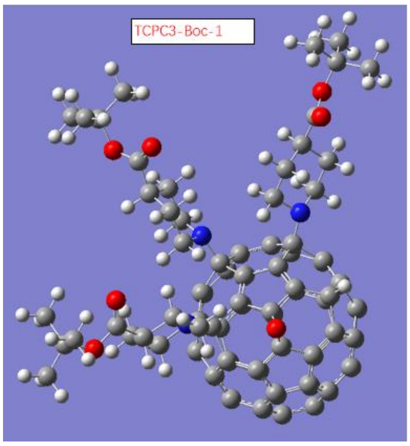

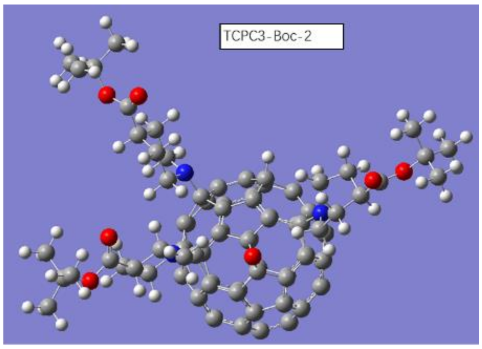

Figure S6: Conformations of different isomers of Boc-protected TAPC-3.

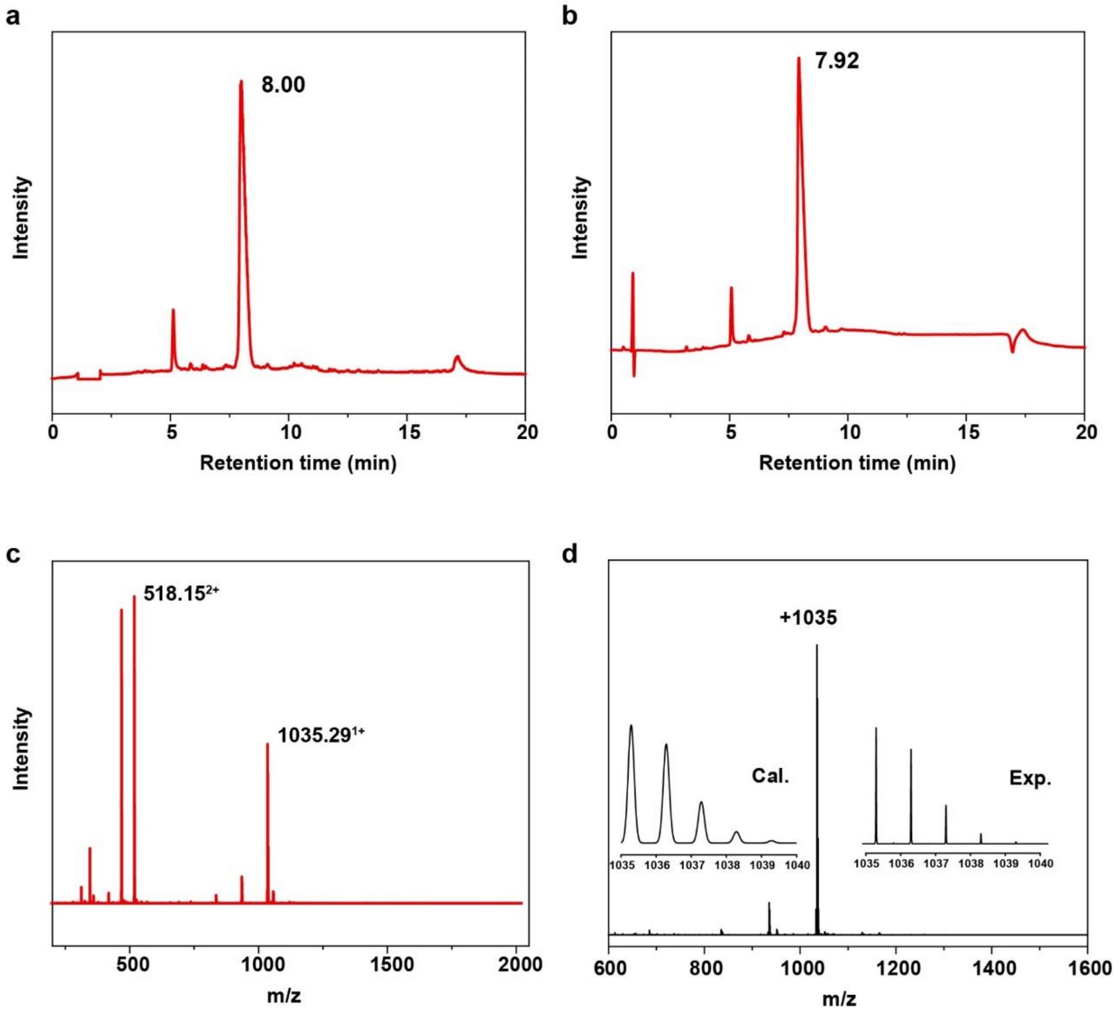

Figure S7: (a) UPLC-TIC detection, (b) UPLC-UV detection, and (c) molecular ionic peaks of TAPC-3. (d) ESI mass spectra of TAPC-3. Insets show the calculated and experimental isotope distribution patterns, respectively.

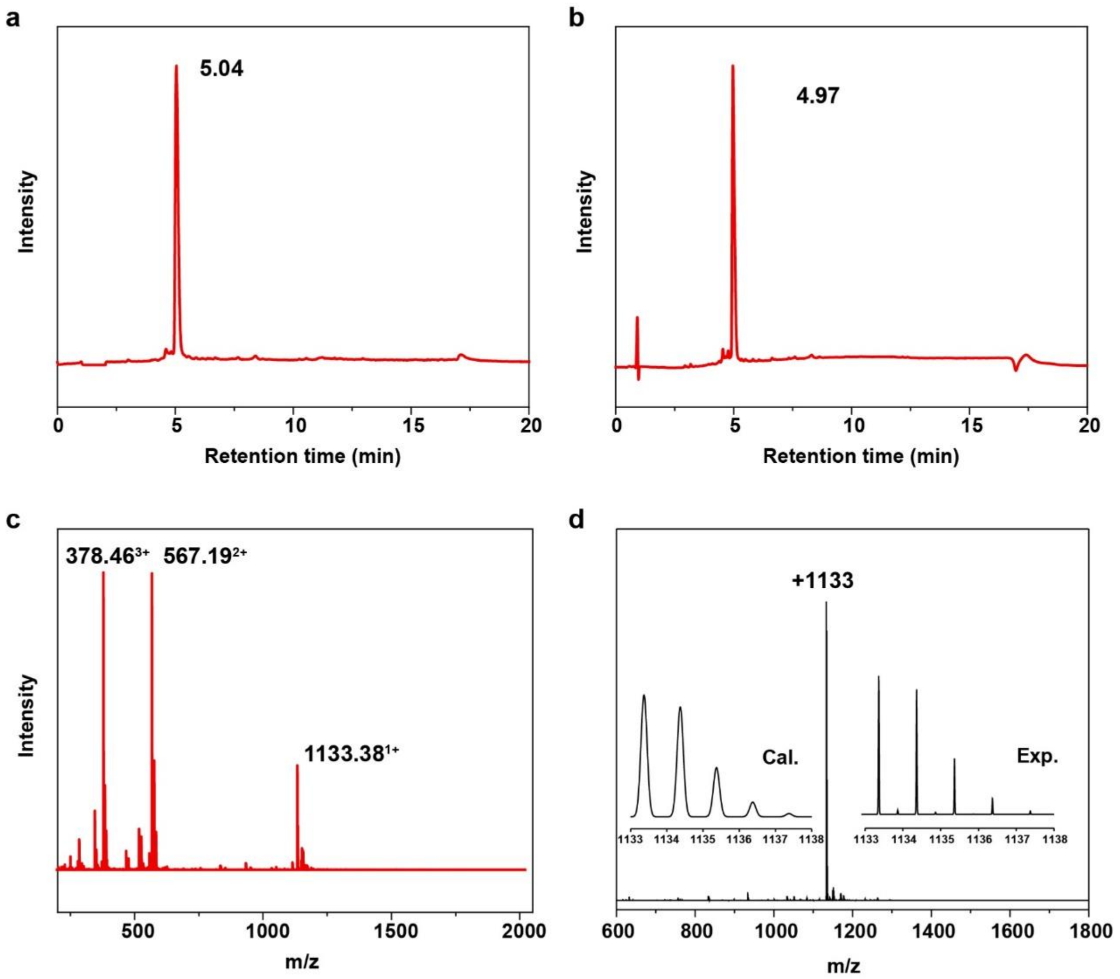

Figure S8: (a) UPLC-TIC detection, (b) UPLC-UV detection, and (c) molecular ionic peaks of TAPC-4. (d) ESI mass spectra of TAPC-4. Insets show the calculated and experimental isotope distribution patterns, respectively.

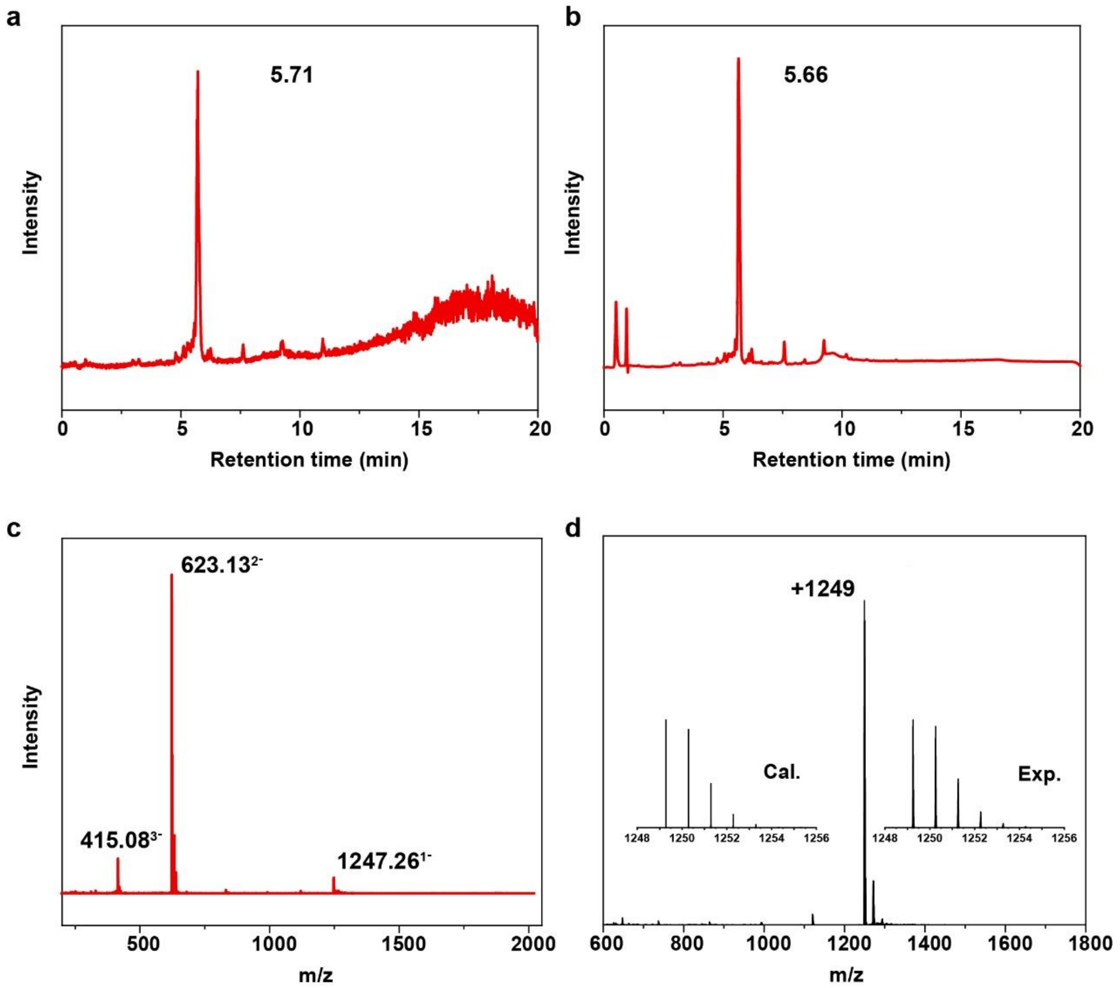

Figure S9: (a) UPLC-TIC detection, (b) UPLC-UV detection, and (c) molecular ionic peaks of TCPC-4. (d) ESI (+) mass spectra of TCPC-4. Insets show the calculated and experimental isotope distribution patterns, respectively.

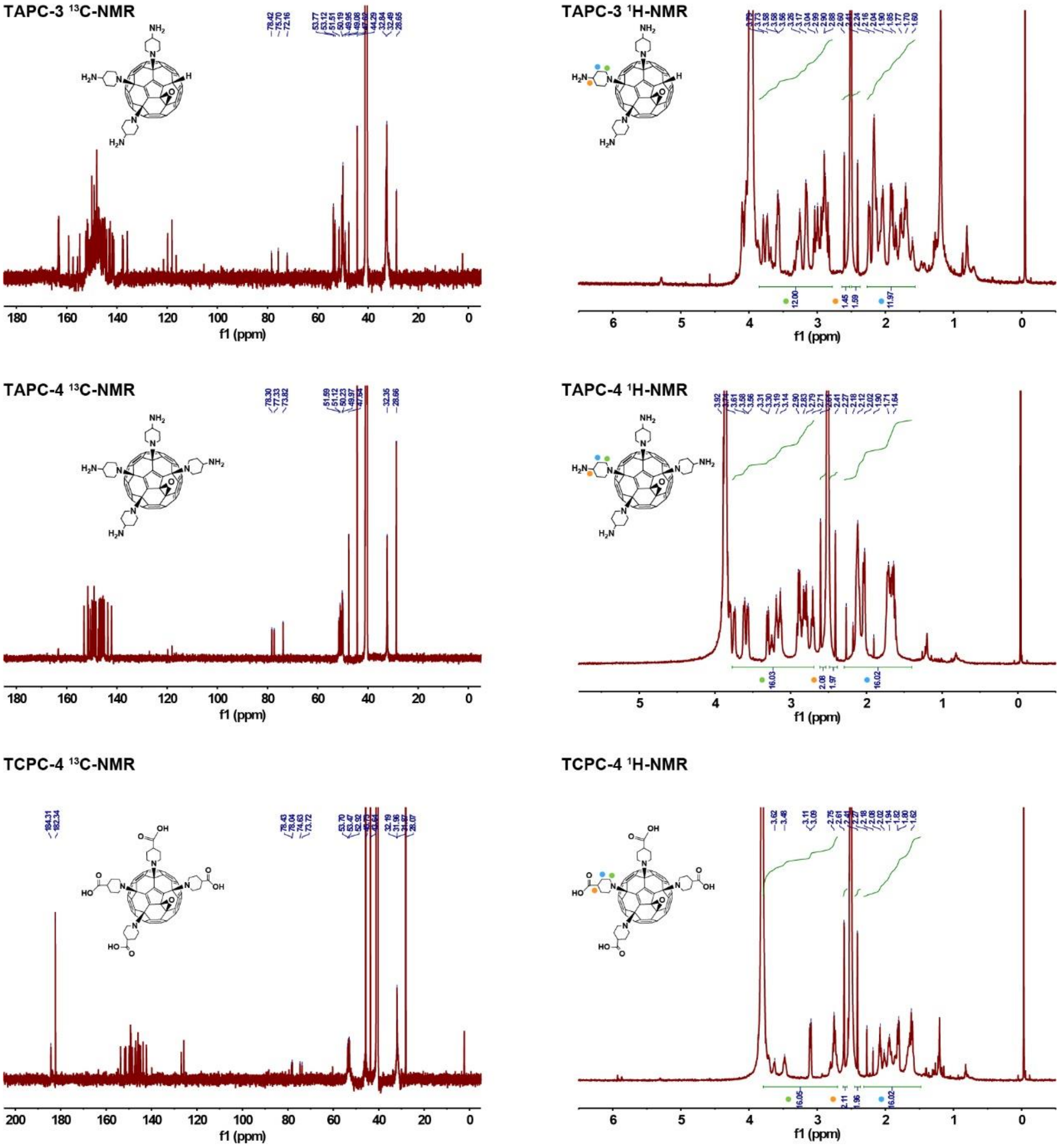

Figure S10: The ^{13}C-NMR and ^{1}H-NMR spectrum of TAPC-3, TAPC-4, and TCPC-4.

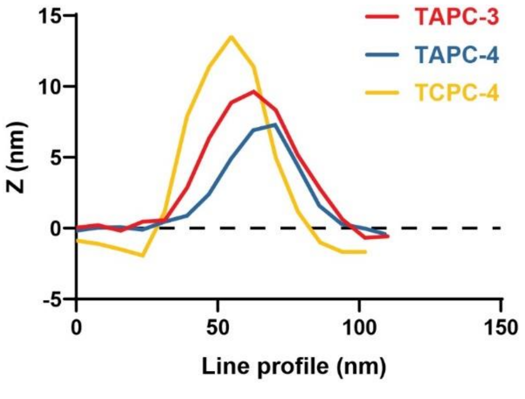

Figure S11: Profile analysis of the lines in Figure 1c.

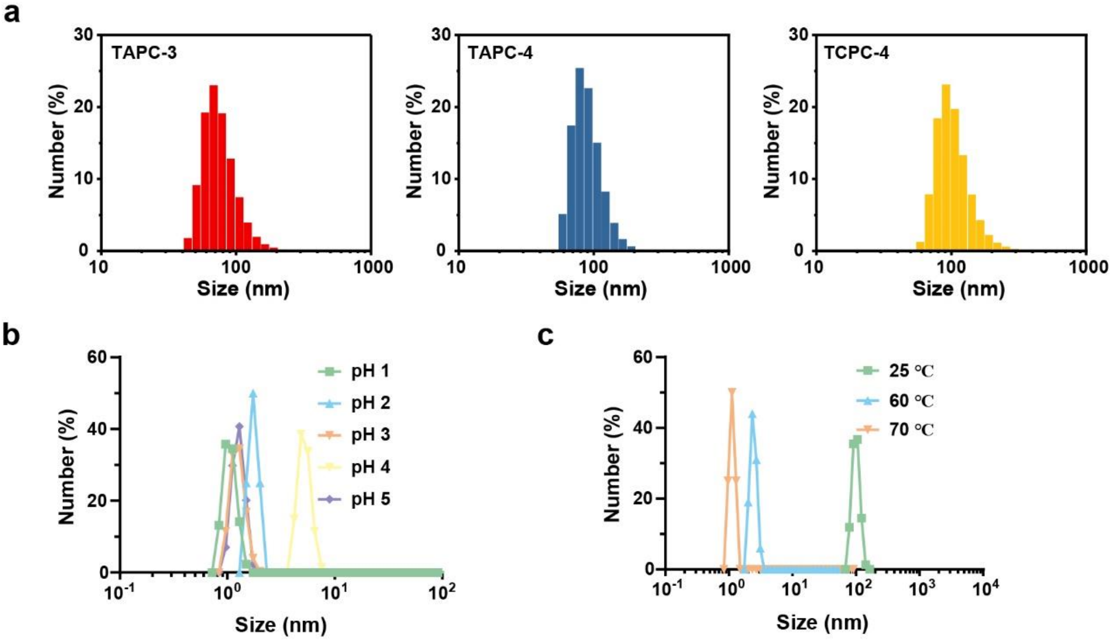

Figure S12: (a) Hydrodynamic size distribution of TAPC-3, TAPC-4, and TCPC-4. Hydrodynamic size distribution of TAPC-4 at (b) different pH and (c) different temperatures.

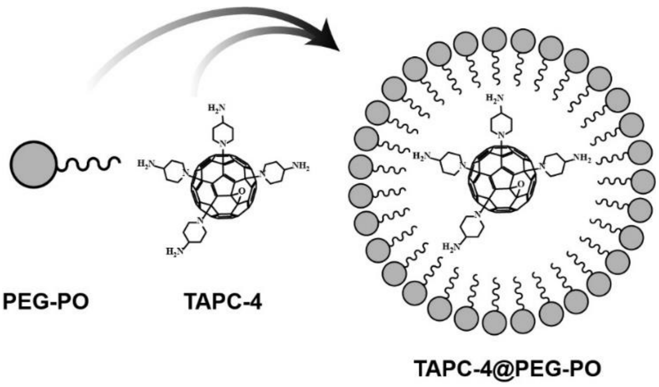

Figure S13: Schematic diagram of PEG-PO coated TAPC-4.

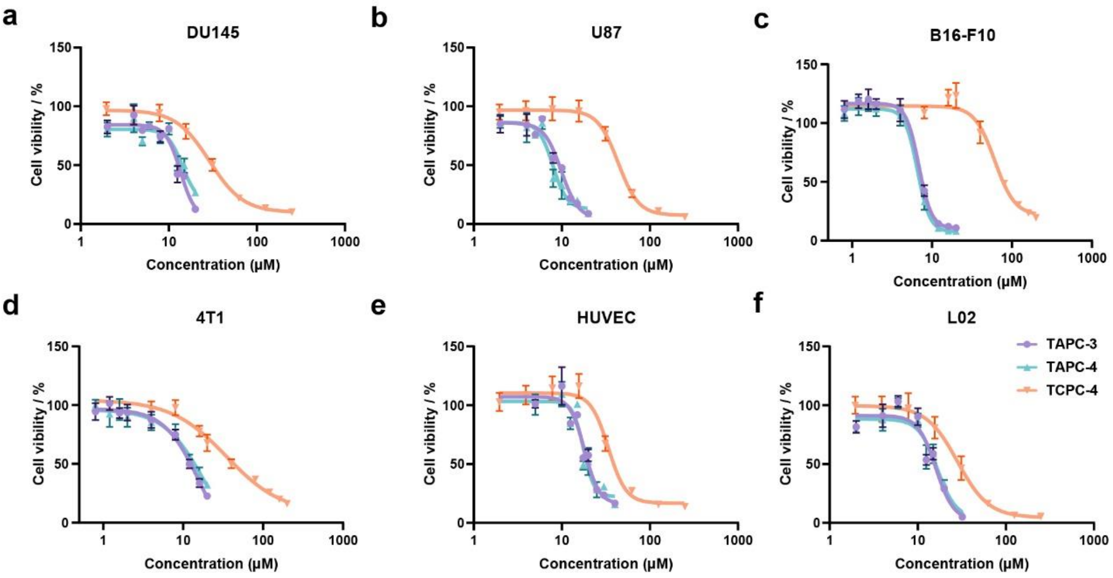

Figure S14: Cell viabilities of DU145, U87, 4T1, B16-F10, L02, and HUVEC cells treated with

TAPC-3, TAPC-4, and TCPC-4 at gradient concentrations for 24 h, respectively (n=6).

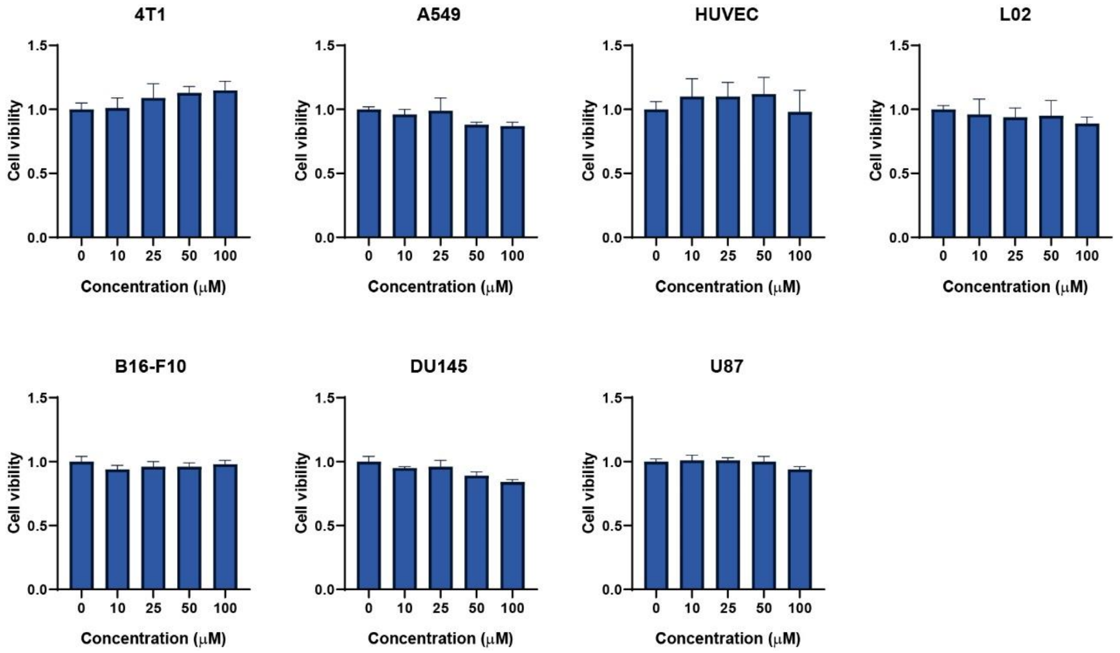

Figure S15: Cell viabilities of DU145, U87, 4T1, B16-F10, L02, and HUVEC cells treated with cyclodextrin-coated C_{60} at gradient concentrations for 24 h, respectively (n=6).

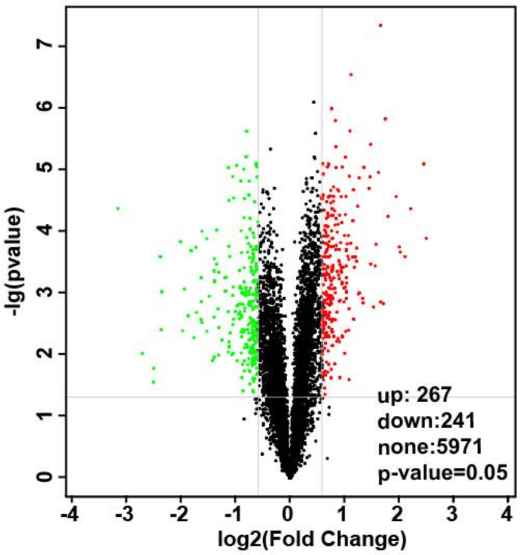

Figure S16: Scatter plots of -$\log_{10}$(P-value) versus $\log_2$(fold change) according to protein expressions. There are 267 significantly up-regulated proteins (P <0.05 and fold change >1.5) represented as red, and 241 significantly down-regulated proteins (P <0.05 and fold change <0.67) as green (n=3).

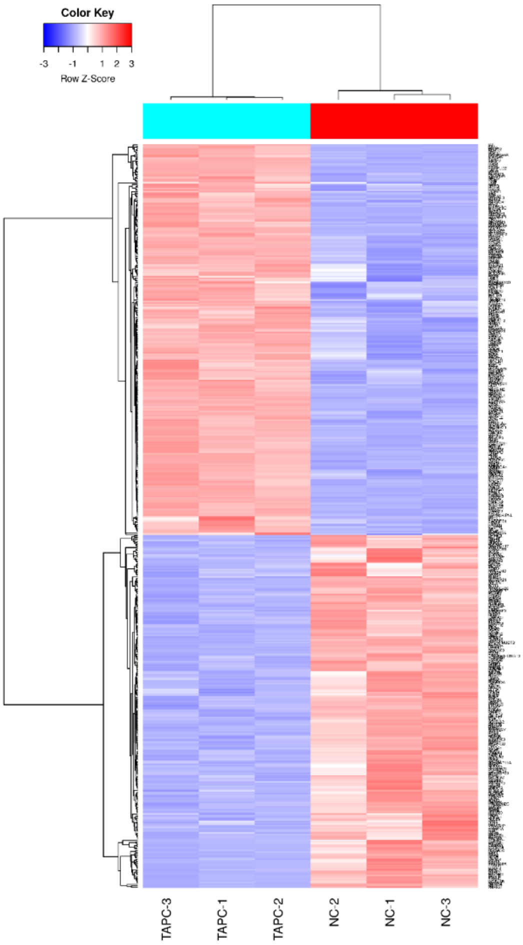

Figure S17: Heat map of comparative proteomic profile. Rows, proteins; columns, samples.

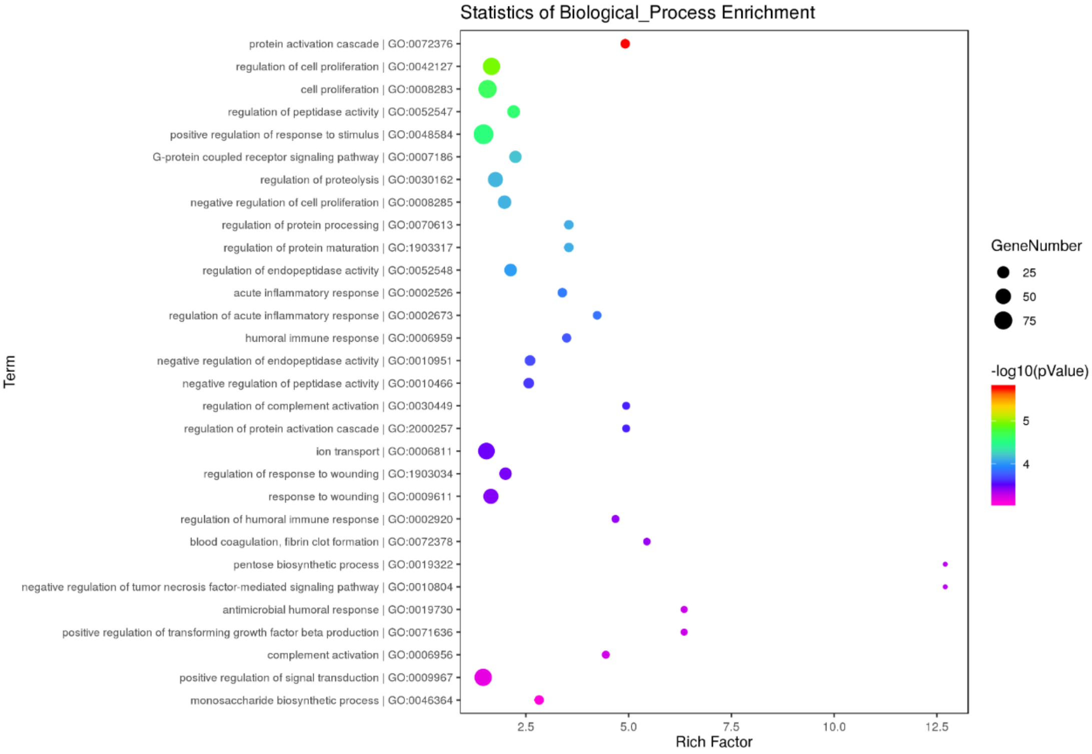

Figure S18: The top 30 significantly enriched GO biological process terms. Bubble diameter represents the number of genes. The bubble color represents the $–\log_{10}$(P-value).

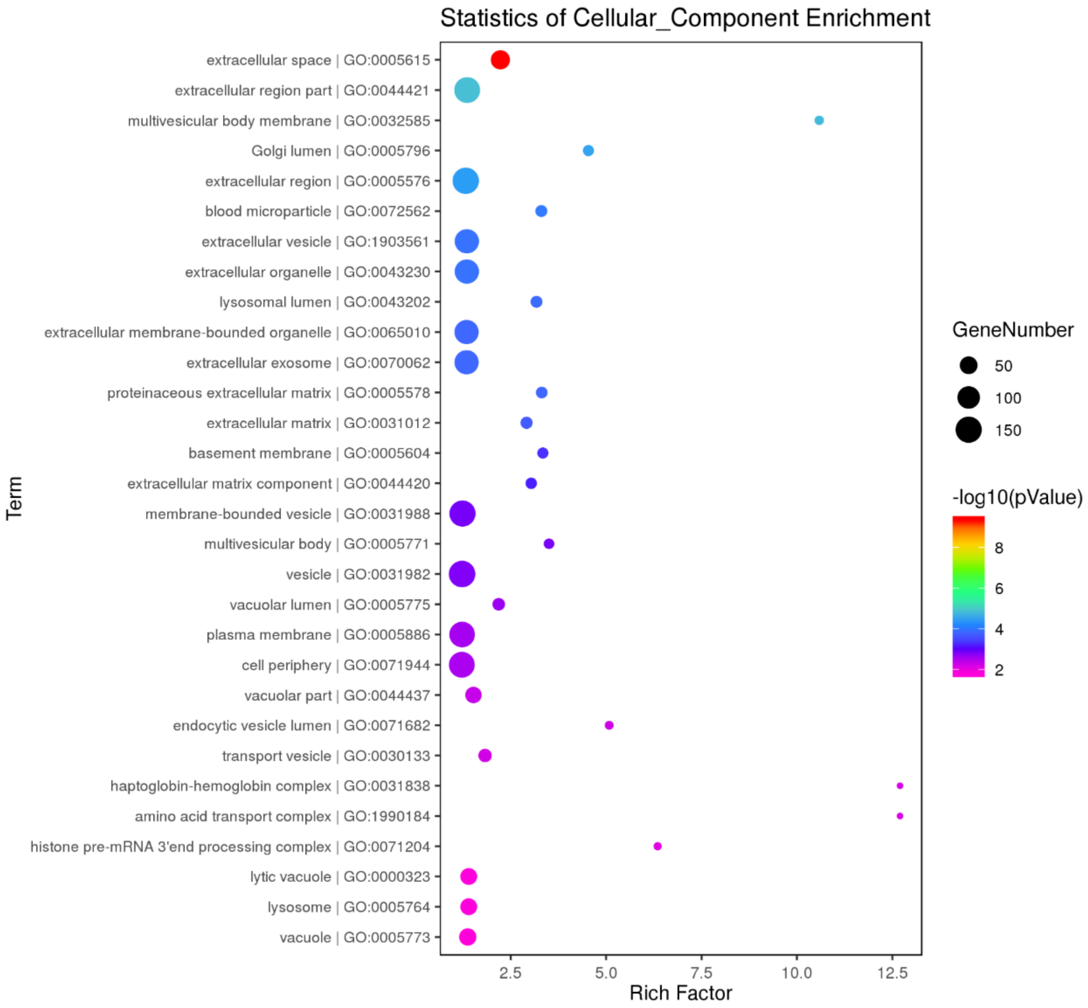

Figure S19: The top 30 significantly enriched GO cellular component terms. Bubble diameter represents the number of genes. The bubble color represents the $–\log_{10}$(P-value).

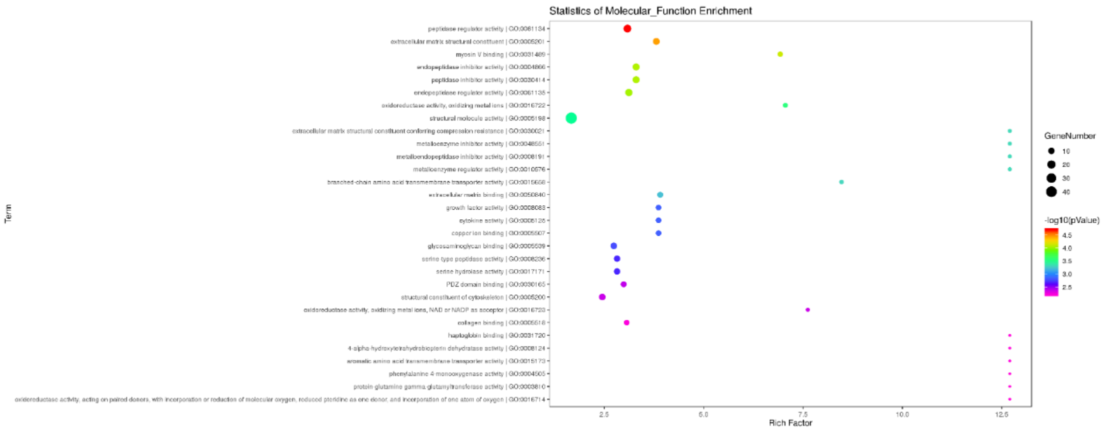

Figure S20: The top 30 significantly enriched GO molecular function terms. Bubble diameter represents the number of genes. The bubble color represents the $–\log_{10}$(P-value).

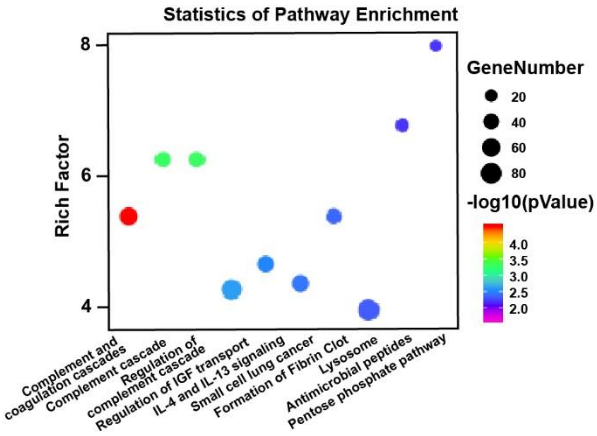

Figure S21: KEGG enrichment analysis of significantly different genes. Significantly the top 10 pathways with the lowest p-value were listed. Bubble diameter represents the number of genes. The bubble color represents the $-\log_{10}$(P-value).

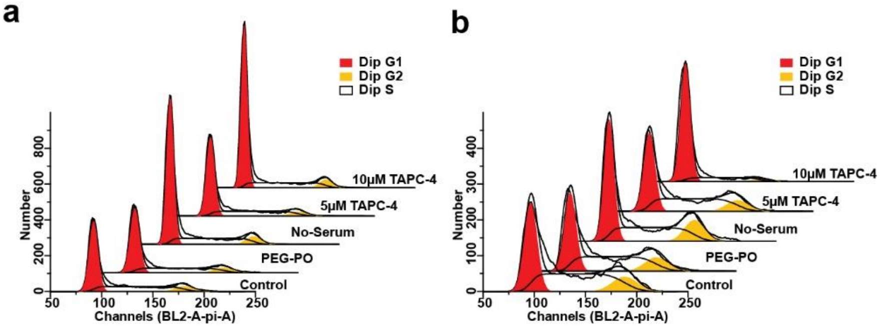

Figure S22: Quantification of (a) A549 and (b) DU145 cells in G0/G1, S, and G2 phase after TAPC-4 treatment or under serum-starved conditions (starvation).

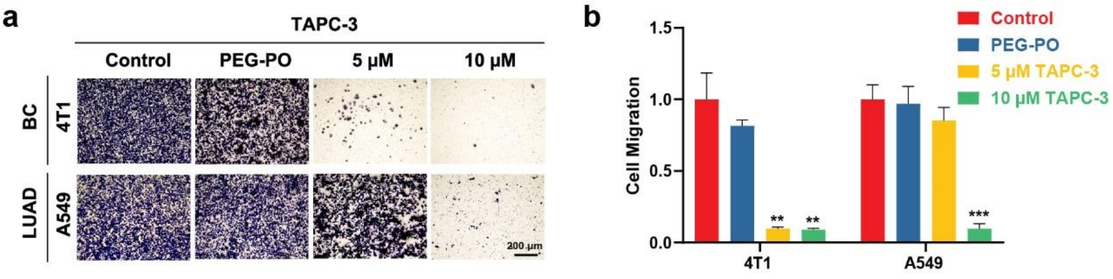

Figure S23: (a-b) Representative images and quantification of migrated cells after TAPC-3 treatment. Cell migration was examined using transwell cell culture chambers. LUAD: lung adenocarcinoma, BC: breast cancer (n=4). (Mean ± SEM; Student's t-test, $^{**}p < 0.01$ and $^{***}p < 0.001$)

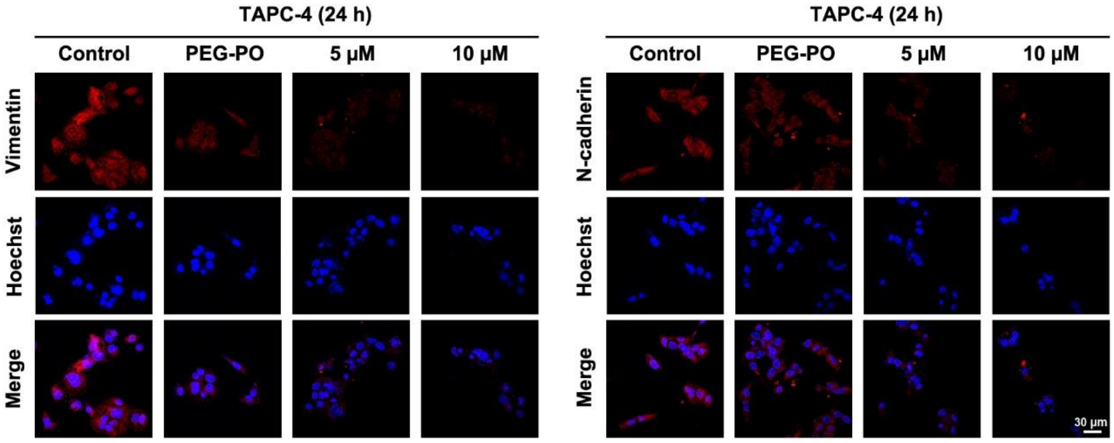

Figure S24: Representative immunofluorescence images of EMT markers (N-cadherin and Vimentin) in DU145 cell lines treated with TAPC-4 for 24 h (n=3).

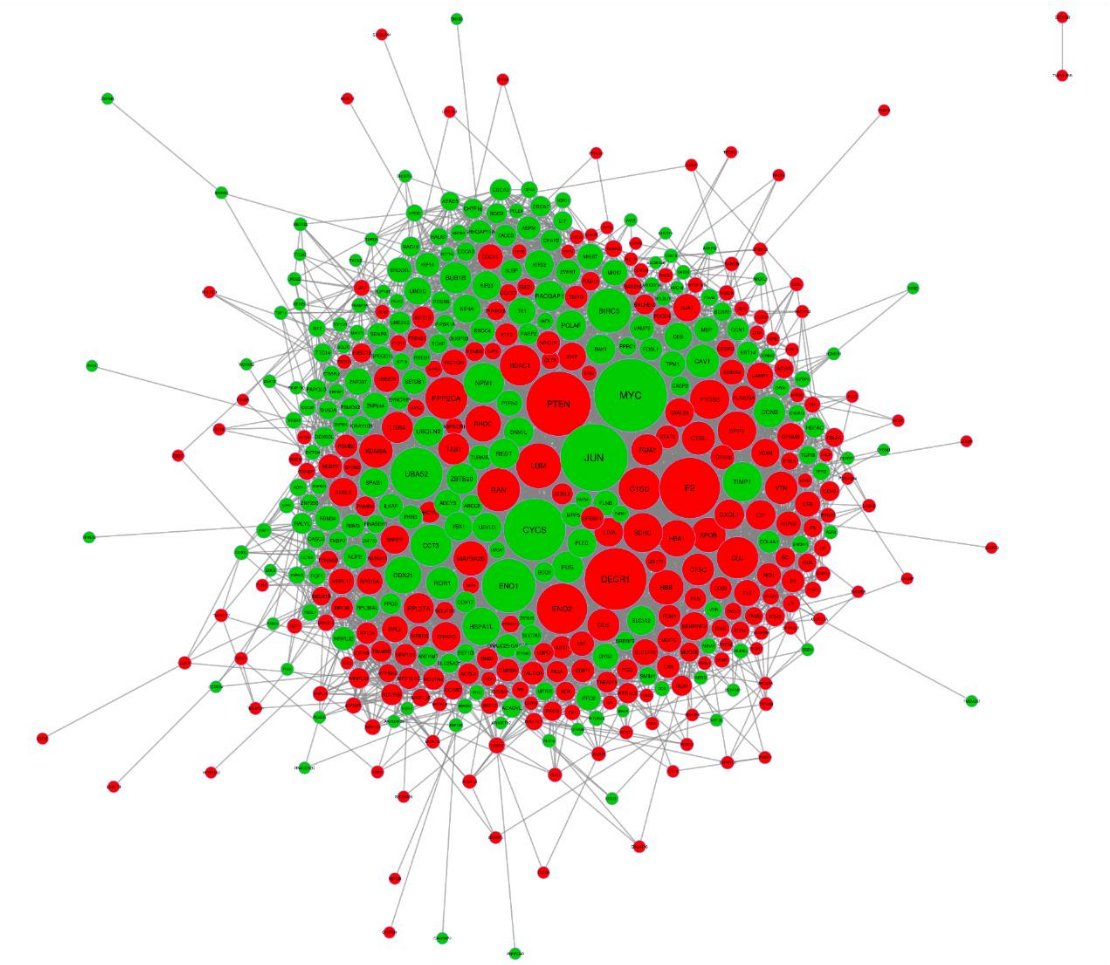

Figure S25: The protein-protein interactions of all significantly changed proteins were analyzed with STRING (http://www.string-db. org).

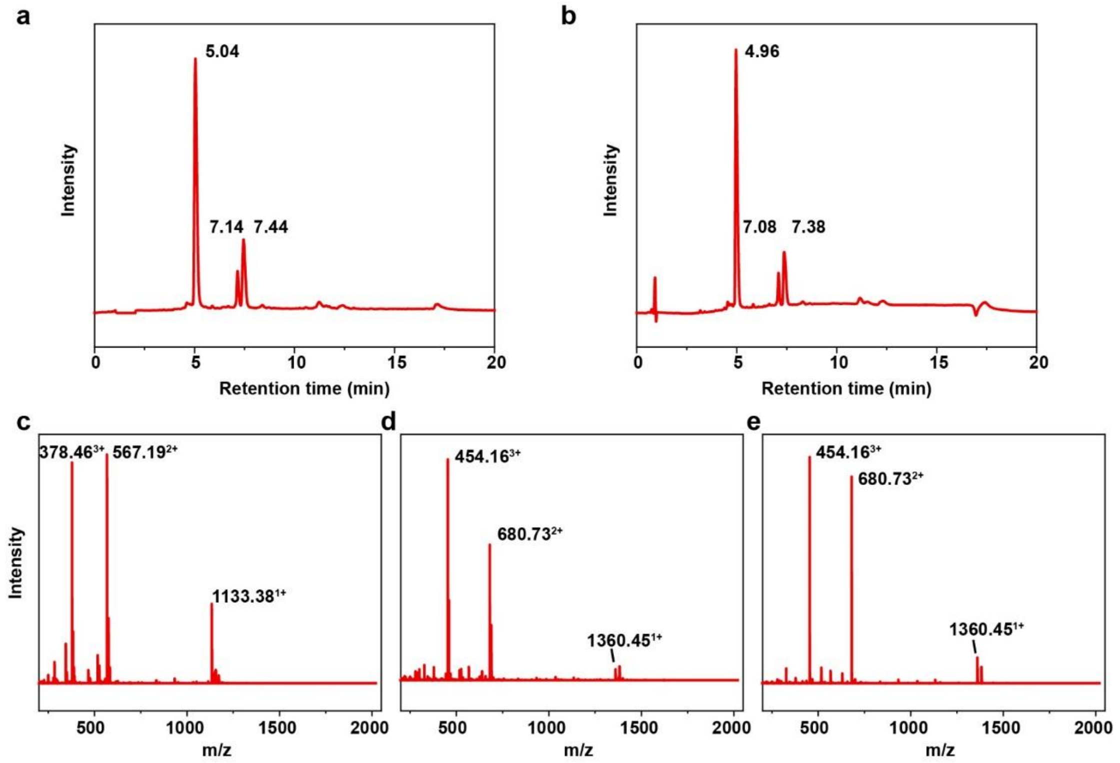

Figure S26: (a) UPLC-TIC detection and (b) UPLC-UV detection of biotinylated TAPC-4. (c) Mass spectrum of TAPC-4 (retention time: 5.0 min). (d-e) Mass spectra of two isomers of biotinylated TAPC-4 (retention time: 7.1 and 7.4 min).

Table S2: Energy calculation for different isomers of biotinylated TAPC-4. Heats of formations (ΔHF); highest occupied molecular orbital (HOMO); lowest unoccupied molecular orbital (LUMO).

Name	Relative ΔHF (kcal/mol)	LUMO-HOMO gap (kcal/mol)
TAPC4-Bio-1	-0.152861314	67.70
TAPC4-Bio-2	0	68.07

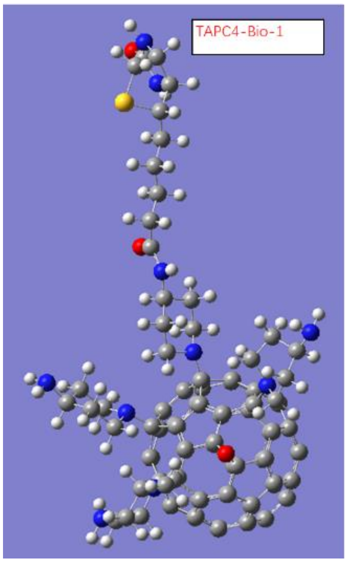

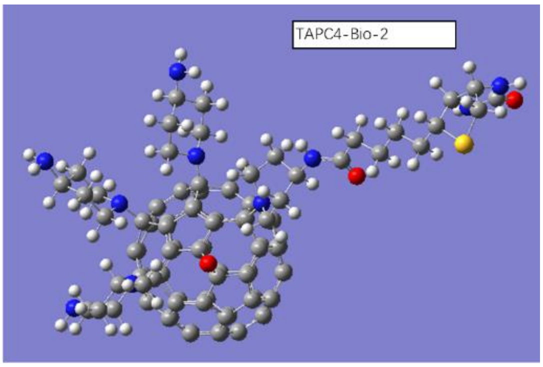

Figure S27: Conformation of different isomers of biotinylated TAPC-4.

Table S3: Top 10 proteins with the highest MS score in each band.

Band ID	Protein IDs	Protein names	Gene names	Score
Band 1	P35579	Myosin-9	MYH9	323.31
	P35580	Myosin-10	MYH10	323.31
	Q01082	Spectrin beta chain, non-erythrocytic 1	SPTBN1	323.31
	P15924	Desmoplakin	DSP	323.31
	O75369	Filamin-B	FLNB	323.31
	P49327	Fatty acid synthase	FASN	323.31
	Q13085	Acetyl-CoA carboxylase 1; Biotin carboxylase	ACACA	323.31
	Q13813	Spectrin alpha chain, non-erythrocytic 1	SPTAN1	323.31
	Q14980	Nuclear mitotic apparatus protein 1	NUMA1	323.31
	Q60FE5	Filamin-A	FLNA	323.31
Band 2	P08238	Heat shock protein HSP 90-beta	HSP90AB1	323.31
	P13639	Elongation factor 2	EEF2	323.31
	P55072	Transitional endoplasmic reticulum ATPase	VCP	323.31
	A0A087WTP3	Far upstream element-binding protein 2	KHSRP	323.31
	Q16891	MICOS complex subunit MIC60	IMMT	323.31
	Q8WUM4	Programmed cell death 6-interacting protein	PDCD6IP	323.31
	P13010	X-ray repair cross-complementing protein 5	XRCC5	316.63
	P47897	Glutamine--tRNA ligase	QARS	310.98
	Q14974	Importin subunit beta-1	KPNB1	270.16
	Q01813	ATP-dependent 6-phosphofructokinase, platelet type	PFKP	267.44
Band 3	P08670	Vimentin	VIM	323.31
	P00352	Retinal dehydrogenase 1	ALDH1A1	323.31
	P11413	Glucose-6-phosphate 1-dehydrogenase	G6PD	323.31
	P30838	Aldehyde dehydrogenase, dimeric NADP-preferring	ALDH3A1	316.57
	P30101	Protein disulfide-isomerase A3	PDIA3	301.72
	P06576	ATP synthase subunit beta, mitochondrial	ATP5B	293.76
	Q02790	Peptidyl-prolyl cis-trans isomerase FKBP4	FKBP4	271.74
	P68363	Tubulin alpha-1B chain	TUBA1B	254.3
	Q9Y230	RuvB-like 2	RUVBL2	251.77
	P50395	Rab GDP dissociation inhibitor beta	GDI2	243.4
Band 4	P05783	Keratin, type I cytoskeletal 18	KRT18	323.31
	P60709	Actin	ACTB	323.31
	P60842	Eukaryotic initiation factor 4A-I	EIF4A1	310.34
	P00558	Phosphoglycerate kinase 1	PGK1	287.64
	Q9UNZ2	NSFL1 cofactor p47	NSFL1C	219.18
	Q15008	26S proteasome non-ATPase regulatory subunit 6	PSMD6	216.99
	Q9Y6N5	Sulfide: quinone oxidoreductase, mitochondrial	SQRDL	211.61
	P49411	Elongation factor Tu, mitochondrial	TUFM	208
	Q99536	Synaptic vesicle membrane protein VAT-1 homolog	VAT1	201.97

	P62195	26S protease regulatory subunit 8	PSMC5	196.99
Band 5	P07355	Annexin A2; Annexin;Putative annexin A2-like protein	ANXA2; ANXA2P2	314.42
	A0A5F9ZHM4	L-lactate dehydrogenase B chain; L-lactate dehydrogenase	LDHB	228.84
	Q5TCU3	Tropomyosin beta chain	TPM2	192.95
	P04083	Annexin A1	ANXA1	187.22
	O60218	Aldo-keto reductase family 1 member B10	AKR1B10	172.54
	Q14847	LIM and SH3 domain protein 1	LASP1	148.31
	O00170	AH receptor-interacting protein	AIP	136.38
	J3KPS3	Fructose-bisphosphate aldolase; Fructose-bisphosphate aldolase A	ALDOA	125.15
	P09525	Annexin A4; Annexin	ANXA4	121.21
	P00338	L-lactate dehydrogenase A chain	LDHA	113.38

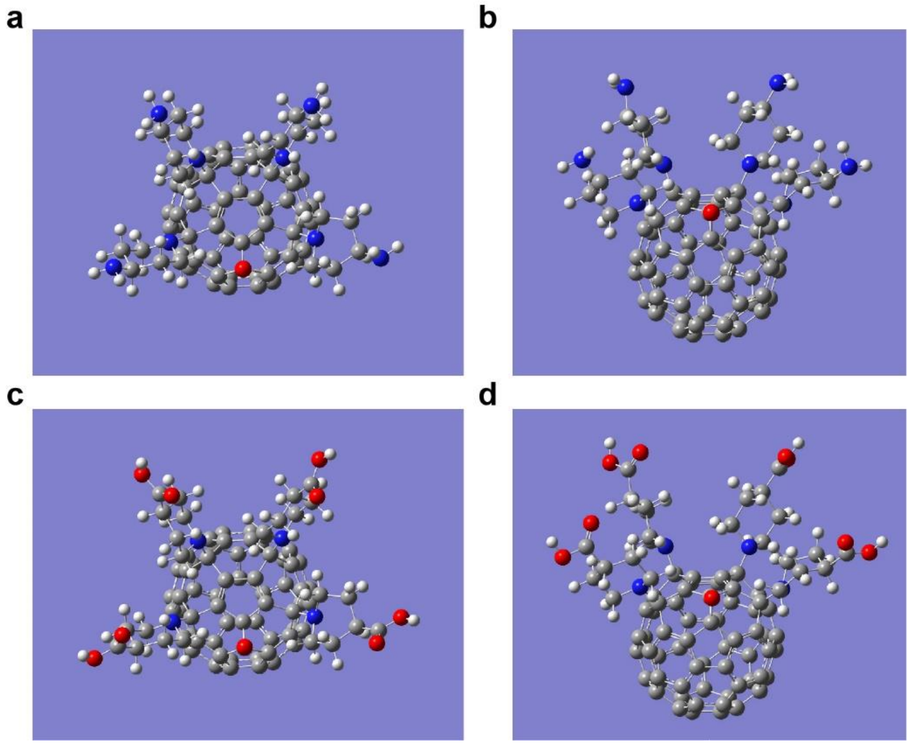

Figure S28: The optimized structure of (a-b) TAPC-4 and (c-d) TCPC-4.

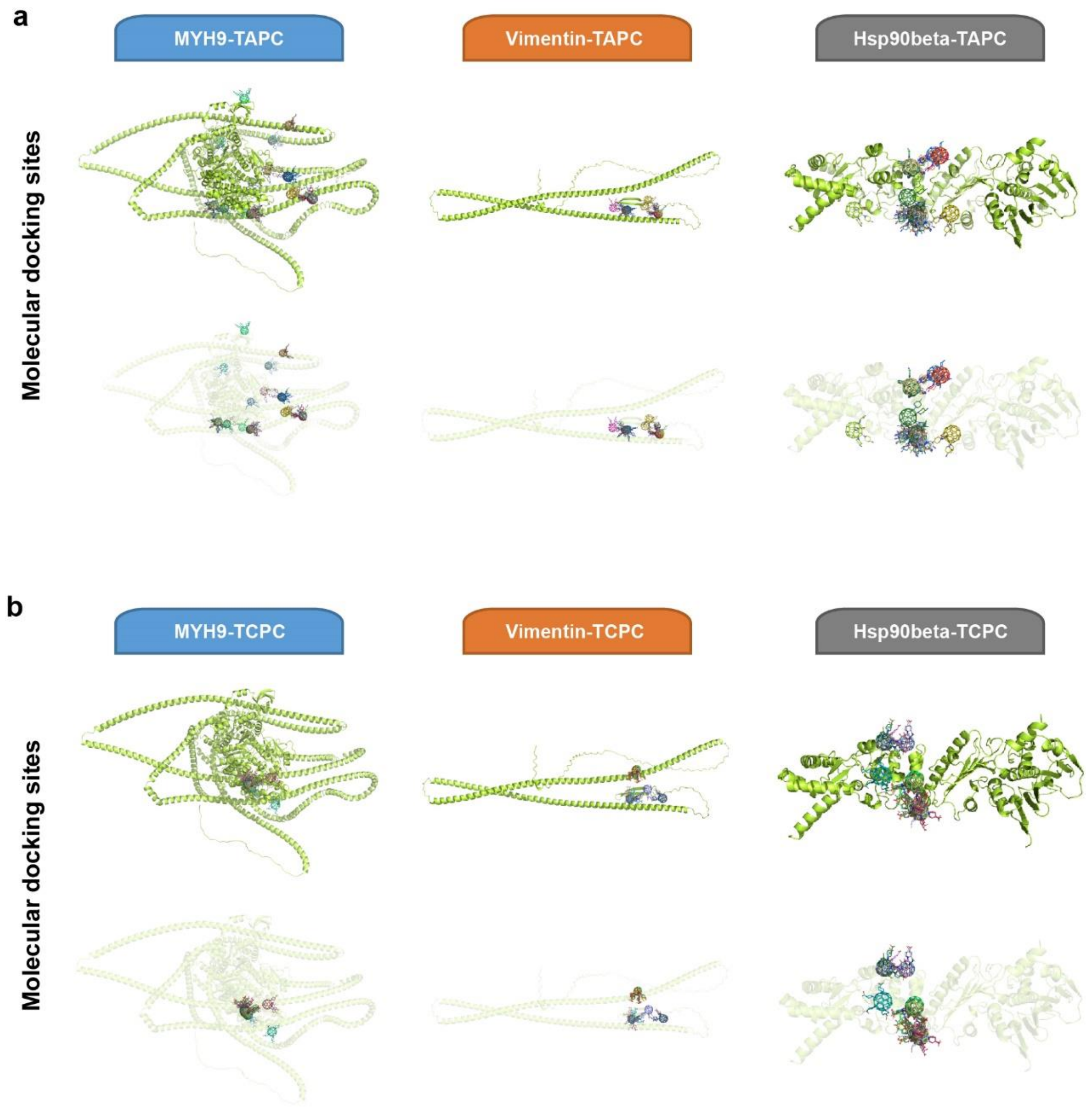

Figure S29: The docking model of ligands (TAPC-4, TCPC-4) to receptors (Hsp90β, vimentin, MYH9).

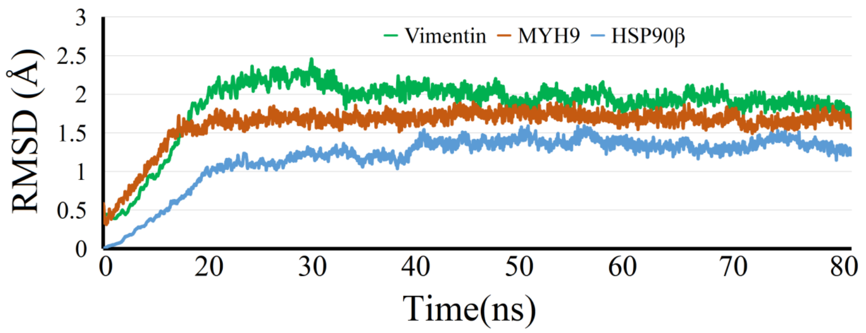

Figure S30: The time dependence of RMSDs for the docked complex of TAPC-4 with Hsp90β, vimentin, and MYH9.

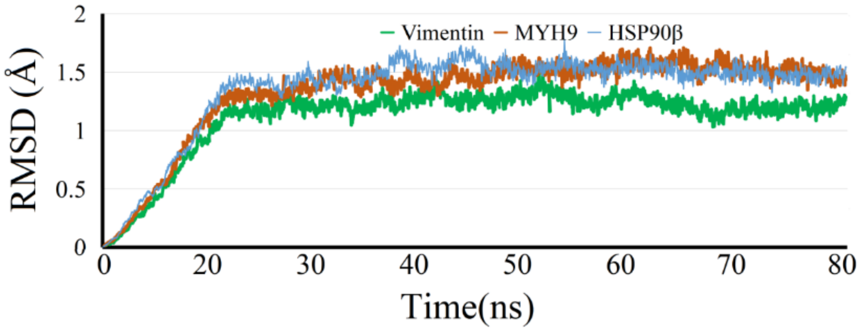

Figure S31: The time dependence of RMSDs for the docked complex of TCPC-4 with Hsp90β, vimentin, and MYH9.

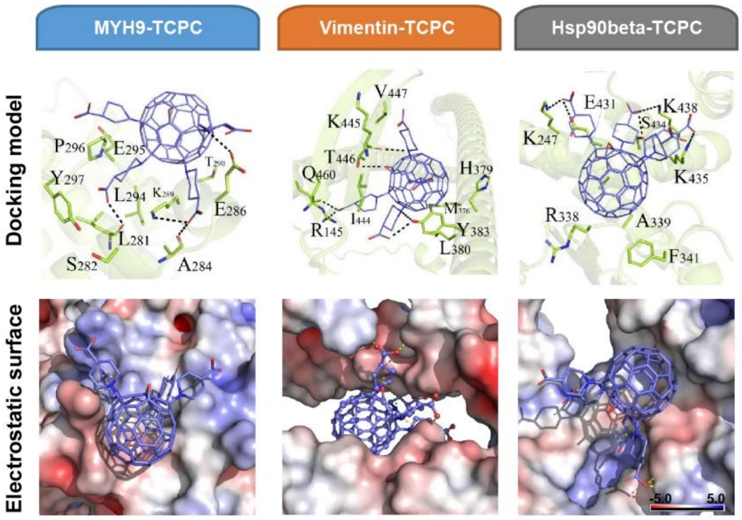

Figure S32: Top: The optimal docking model of TCPC-4 to MYH9, vimentin, and Hsp90β. TAPC-4 and amino acids are shown in blue and green, respectively. Bottom: The binding mode of TCPC-4 in MYH9, vimentin, and Hsp90β. The active site pocket is displayed as an electrostatic surface.

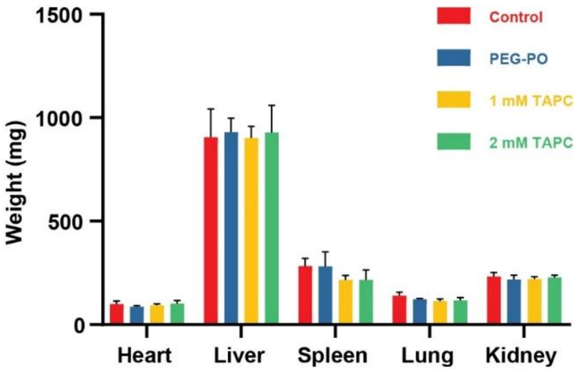

Figure S33: Weight of heart, liver, spleen, lung, and kidney collected on the 14th day.

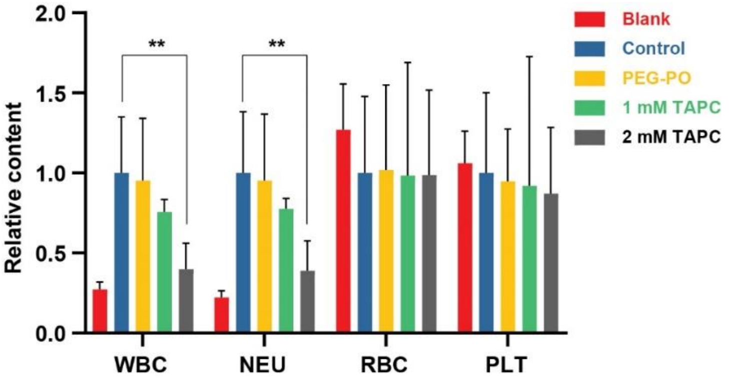

Figure S34: Blood routine analysis on the 14th day.

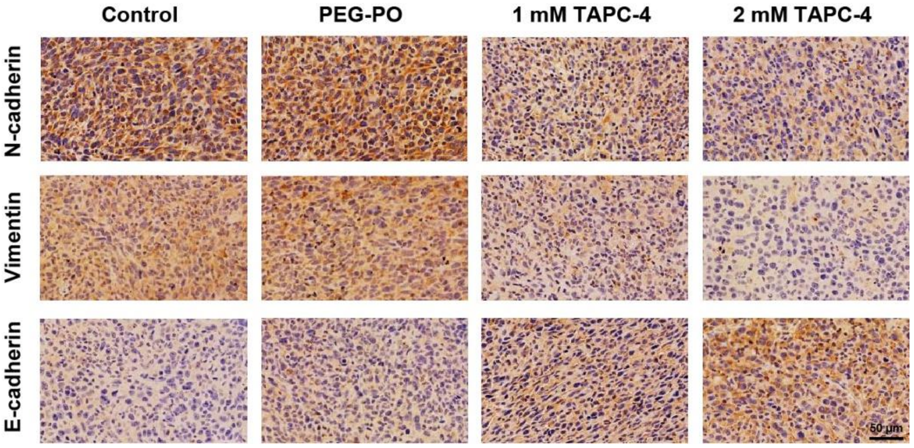

Figure S35: Immunohistochemical staining of N-cadherin, Vimentin, and E-cadherin in tumors. The scale bar is 50 μm.

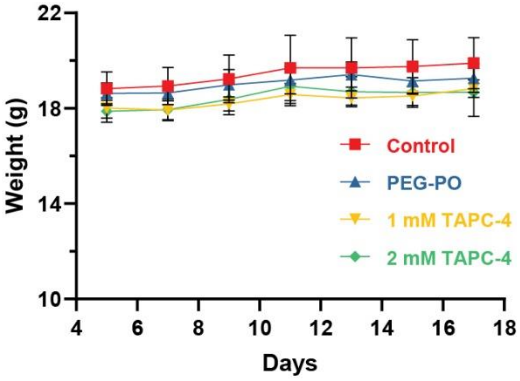

Figure S36: Negligible changes in body weight of the mice (n=7).
